# Metal hydrides: an innovative and challenging conversion reaction anode for lithium-ion batteries

**DOI:** 10.3762/bjnano.6.186

**Published:** 2015-08-31

**Authors:** Luc Aymard, Yassine Oumellal, Jean-Pierre Bonnet

**Affiliations:** 1 Laboratoire de Réactivité et Chimie des Solides - LRCS, UMR CNRS-UPJV 7314, 33 rue Saint-Leu, 80039 Amiens, France; 2 Institut de Chimie et des Matériaux Paris-Est - ICMPE, UMR CNRS-UPEC 7182, 2-8 Rue Henri Dunant, 94320 Thiais, France

**Keywords:** conversion reaction, lithium-ion batteries, metal hydrides

## Abstract

The state of the art of conversion reactions of metal hydrides (MH) with lithium is presented and discussed in this review with regard to the use of these hydrides as anode materials for lithium-ion batteries. A focus on the gravimetric and volumetric storage capacities for different examples from binary, ternary and complex hydrides is presented, with a comparison between thermodynamic prediction and experimental results. MgH_2_ constitutes one of the most attractive metal hydrides with a reversible capacity of 1480 mA·h·g^−1^ at a suitable potential (0.5 V vs Li^+^/Li^0^) and the lowest electrode polarization (<0.2 V) for conversion materials. Conversion process reaction mechanisms with lithium are subsequently detailed for MgH_2_, TiH_2_, complex hydrides Mg_2_MH*_x_* and other Mg-based hydrides. The reversible conversion reaction mechanism of MgH_2_, which is lithium-controlled, can be extended to others hydrides as: MH*_x_* + *x*Li^+^ + *x*e^−^ in equilibrium with M + *x*LiH. Other reaction paths—involving solid solutions, metastable distorted phases, and phases with low hydrogen content—were recently reported for TiH_2_ and Mg_2_FeH_6_, Mg_2_CoH_5_ and Mg_2_NiH_4_. The importance of fundamental aspects to overcome technological difficulties is discussed with a focus on conversion reaction limitations in the case of MgH_2_. The influence of MgH_2_ particle size, mechanical grinding, hydrogen sorption cycles, grinding with carbon, reactive milling under hydrogen, and metal and catalyst addition to the MgH_2_/carbon composite on kinetics improvement and reversibility is presented. Drastic technological improvement in order to the enhance conversion process efficiencies is needed for practical applications. The main goals are minimizing the impact of electrode volume variation during lithium extraction and overcoming the poor electronic conductivity of LiH. To use polymer binders to improve the cycle life of the hydride-based electrode and to synthesize nanoscale composite hydride can be helpful to address these drawbacks. The development of high-capacity hydride anodes should be inspired by the emergent nano-research prospects which share the knowledge of both hydrogen-storage and lithium-anode communities.

## Review

### Introduction

To satisfy the continuously raising need for energy is now a key priority worldwide. The challenge is to obtain environmentally friendly renewable power sources with enhanced electrical energy conversion efficiency at moderate costs. However, these energy sources, such as windmill or solar cells, are intrinsically intermittent and, consequently, need to be associated with efficient energy storage devices in order to provide electricity on demand. With regard to this, lithium-ion (Li-ion) batteries can present an attractive solution, provided that they exhibit sufficient potential and gravimetric/volumetric capacities. Graphite, which is usually used as negative electrode with an intercalation reaction of lithium, is not suitable here due to its intrinsic insufficient specific capacities (370 A·h·kg^−1^, 840 A·h·L^−1^). To overcome these restrictions, new concepts for the negative electrode must be developed, i.e., the Li/graphite intercalation reaction needs to be replaced by either alloying or conversion reactions with lithium. Previously, metal oxides, nitrides, sulfides, phosphides and fluorides were successively investigated as conversion-reaction materials for the negative electrodes of Li-ion batteries [1–4]. In 2008, metal hydrides were proposed for this purpose [5]. Compared to other conversion compounds MgH_2_ exhibits remarkable properties such as the lowest polarization value for conversion electrodes (less than 0.2 V) at an average potential of 0.5 V vs Li^+^/Li^0^ and a high reversible capacity (1480 mA·h·g^−1^ which is four times that of Li/C electrodes). All these properties make MgH_2_ suitable as a material for negative electrodes. MgH_2_ reacts with lithium ions in a reversible lithium-driven conversion reaction generating lithium hydride and magnesium metal: MgH_2_ + 2Li^+^ + 2e^−^ 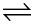 Mg + 2LiH. Moreover, this conversion reaction is not restricted to MgH_2_. It can also be carried out with several different binary and ternary hydrides. The general chemical reaction is then: MH*_x_* + *x*Li^+^ + *x*e^−^ 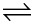 M + *x*LiH.

The purpose of this review is to describe the properties of these metal hydrides properties in the reaction vs Li^+^/Li^0^ (conversion reaction) with a focus on thermodynamics, involved reaction mechanisms, and some key issues to improve the performance of hydride-based electrodes.

### I Conversion reaction of hydrides with lithium ions

#### I.1 Gravimetric and volumetric storage capacity of hydrides

**Figure 1:**
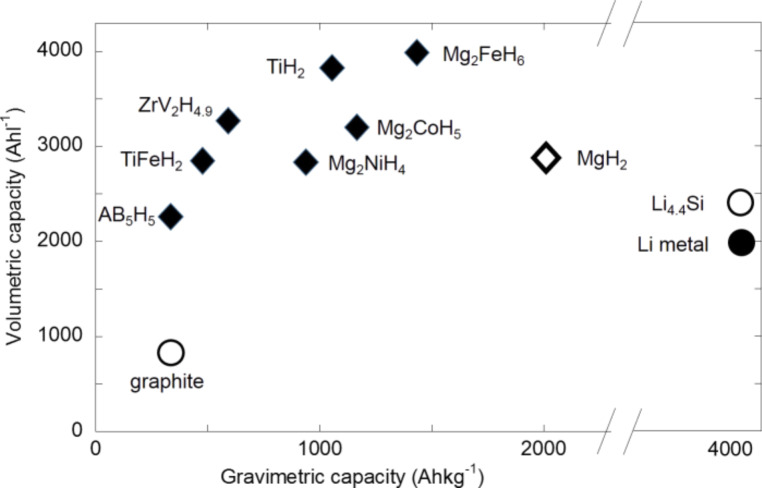
Gravimetric and theoretical volumetric capacities of metals and complex hydrides compared with those of graphite and other materials for negative electrodes. Reproduced with permission from [13]. Copyright 2013 Elsevier.

Figure 1 shows both theoretical gravimetric and volumetric capacities of some binary and ternary hydrides. It can be noticed that the capacities of all hydrides are larger than that of graphite (370 A·h·kg^−1^, 840 A·h·L^−1^). Regarding ternary hydrides, the gravimetric capacities are between 340 A·h·kg^−1^ (LaNi_4_MnH_5_) and 750 A·h·kg^−1^ (ZrV_2_H_4.9_). The highest values are obtained with binary hydrides of light metals, namely 1074 A·h·kg^−1^ and 2038 A·h·kg^−1^ for TiH_2_ and MgH_2_, respectively. Volumetric capacities are above 2000 A·h·L^−1^ for all hydrides, for instance, 2298 A·h·L^−1^, 2878 A·h·L^−1^, 3815 A·h·L^−1^ for LaNi_4_MnH_5,_ MgH_2_ and TiH_2_, respectively. Complex hydrides based on Mg follow this general trend of hydrides (i.e., capacities larger than graphite with Mg_2_NiH_4_: 963 A·h·kg^−1^, 2822 A·h·L^−1^; Mg_2_CoH_5_: 1191 A·h·kg^−1^, 3200 A·h·L^−1^; Mg_2_FeH_6_: 1456 A·h·kg^−1^, 3995 A·h·L^−1^). These large capacities render hydrides as good candidate material for negative electrodes in lithium-ion batteries for stationary as well as mobile applications for which the volumetric capacity plays a key role.

#### I.2 Thermodynamics of hydrides

After experimental results on the conversion reaction with MgH_2_/Li [5–6] were reported, other systems that could be involved in the electrochemical conversion process were addressed from thermodynamic rules. The general reaction between hydride and lithium is given in Equation 1, where MH*_x_* is the hydride, M the metal or the intermetallic phase, and *x* the number of hydrogen atoms. Under standard conditions (*p* = 1 atm and *T* = 298 K), the Gibbs free energy of the reaction in Equation 1, Δ_r_*G* in kJ·mol^−1^, can be calculated from the values of Gibbs free energy of formation of MH*_x_* and LiH (Δ_f_*G*°_298_(LiH) and Δ_f_*G*°_298_(MH*_x_*). It corresponds to the sum of reactions (Equation 2 + Equation 3) given as:

[2]
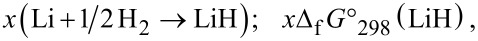


[3]
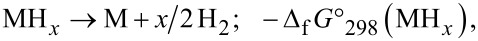


[1]
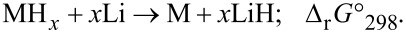


The Gibbs free energy of the reaction in Equation 1 is:


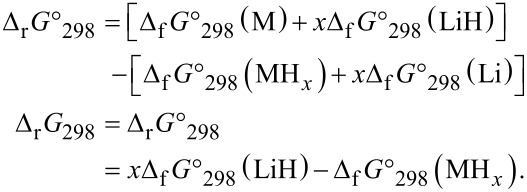


According to the thermodynamics rules, the reaction is feasible for Δ_r_*G*°_298_ ≤ 0 and spontaneous for Δ_r_*G*°_298_ < 0.

Given the fact that the formation of LiH independent from the type of hydride MH*_x_* (common reaction product), another simple criterion to predict the possibility of the conversion process is the Gibbs free energy of formation of the hydride MH*_x_* divided by *x*. This value must be above that of LiH (Δ_f_*G*°_298_(MH*_x_*)/*x* > Δ_f_*G*°_298_(LiH)). The knowledge of reaction Gibbs free energy allows for the evaluation of the electromotive force (emf) and of the equilibrium potential of the cell, *E*, by using the Nernst law: Δ_r_*G*° = −*x*·*E*·*F* (*F*: Faraday constant, *x*: number of electrons involved in the reaction). The equilibrium potential of the cell is deduced from the half reaction (Equation 4 and Equation 5) and the sum reaction (Equation 6).

[4]
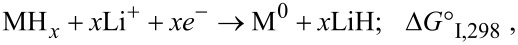


[5]
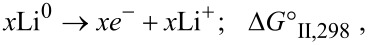


[6]
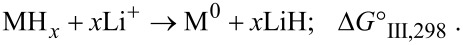


Hess’s law gives:


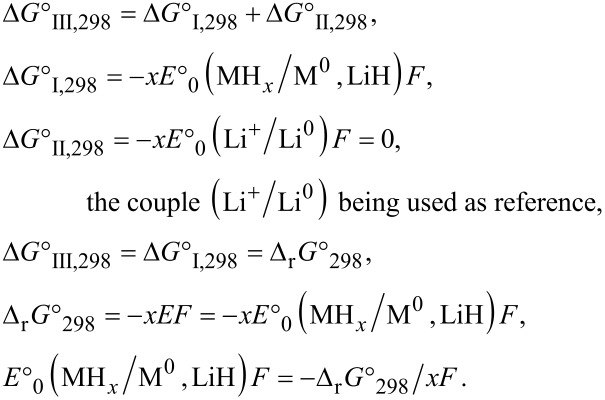


With a lithium activity *a*(Li) = 0, and a lithium ion concentration [Li^+^] = 1 M ([Li^+^] inside the electrolyte), the equilibrium potential of the cell (*E*_eq_) is:


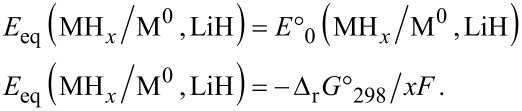


This calculation was applied for different binary and ternary hydrides used to represent the different intermetallic families. The HSC database [7] and literature data [8] were used to obtain the Δ_f_*H*, Δ_f_*G* and Δ_f_*S* values.

**Figure 2:**
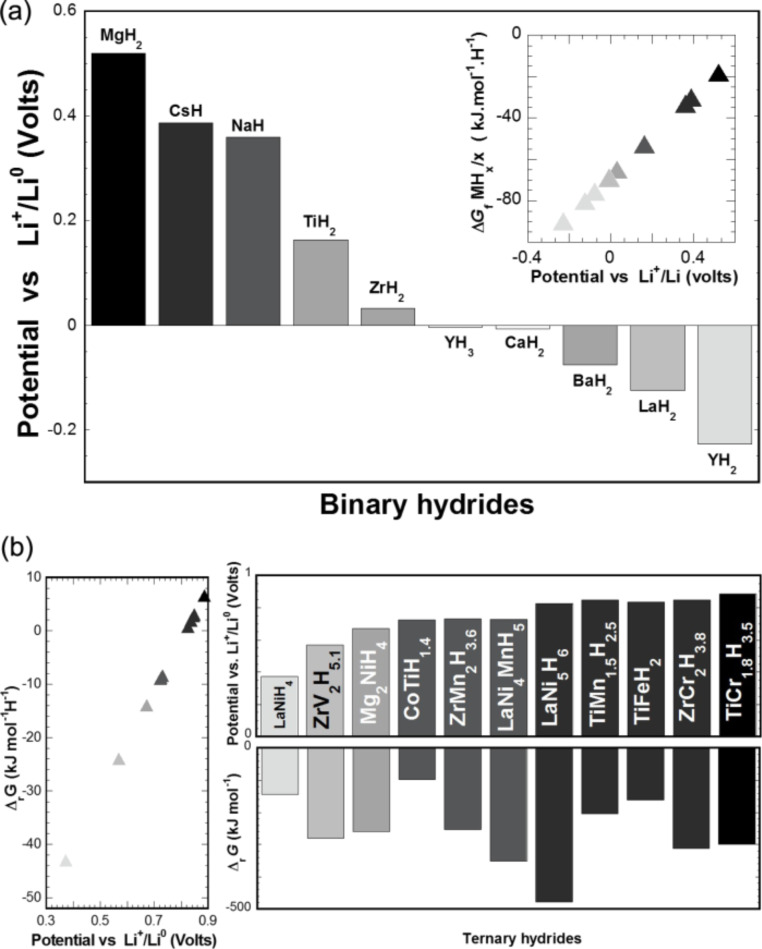
Theoretical equilibrium potential for the MH*_x_*/Li cell vs Li^+^/Li^0^. a) For binary hydrides M = Y, La, Ba, Ca, Zr, Ti, Na, Cs, Mg. Inset: Gibbs free formation enthalpy of these hydrides as a function of the equilibrium potential. b) For ternary hydrides Mg_2_NiH_4_, LaNiH_4_, CoTiH_1.4_, TiFeH_2_, ZrV_2_H_5.1_, ZrCr_2_H_3.8_, ZrMn_2_H_3.6_, TiMn_1.5_H_2.5_, TiCr_1.8_H_3.5_, LaNi_5_H_6_, LaNi_4_MnH_5_ [11].

**I.2.1 Binary hydrides:** Figure 2a shows the equilibrium potential of the MH*_x_*/Li cell for different binary hydrides M = Y, La, Ba, Ca, Zr, Ti, Na, Cs, Mg. The potential versus Li^+^/Li^0^ is positive for Zr, Ti, Na, Cs and Mg and negative for Y, La, Ba and Ca hydrides. It can be clearly concluded from this figure that the conversion reaction is favorable, from a thermodynamics point of view, for Zr, Ti, Na, Cs and Mg hydrides and not possible for Y, La, Ba, Ca. The Gibbs free enthalpy of formation (divided by the amount of substance of hydrogen) of these hydrides is smaller than that of LiH (inset Figure 2a).

**I.2.2 Ternary hydrides:** In the case of ternary hydrides, equilibrium potentials of the MH*_x_*/Li cells were evaluated for different representative compounds AB*_x_* of intermetallic families (with *x* = 0.5, 1, 2 and 5) and their corresponding hydrides (Mg_2_NiH_4_, LaNiH_4_, CoTiH_1.4_, TiFeH_2_, ZrV_2_H_5.1_, ZrCr_2_H_3.8_, ZrMn_2_H_3.6_, TiMn_1.5_H_2.5_, TiCr_1.8_H_3.5_, LaNi_5_H_6_, LaNi_4_MnH_5_). Figure 2b (lower part) shows that the Gibbs free enthalpy of formation value for all these ternary hydrides (per mole hydrogen) is above that of LiH. For these hydrides the equilibrium potential of the cell is positive (Figure 2b) and the conversion reaction can be achieved. Equilibrium potentials are in the range of 0.3–1.0 V vs Li^+^/Li^0^, which is suitable for a negative electrode in Li-ion batteries. The equilibrium potential of the cell can be adjusted for different AB*_x_* intermetallic families by varying the site substitutions of A and B [8]. In fact, the plateau pressure of hydride correlates with the lattice cell volume, allows one to change the thermodynamic stability of the hydrides, especially for the families AB_5_ and AB_2_. Depending of the nature of the hydrides a wide range of hydrogen sorption temperatures from −40 to 300 °C gives another argument to a tailor a negative electrode for the desired applications [9].

#### I.3 Comparison between thermodynamic prediction and experience

**Figure 3:**
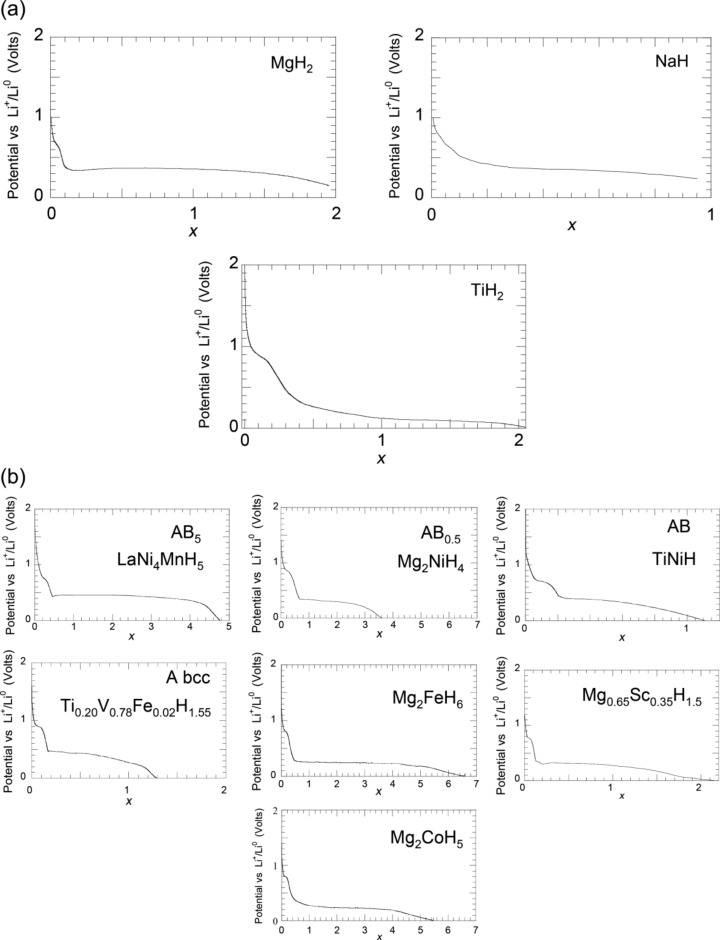
Potentials vs Li^+^/Li^0^ of MH*_x_*/Li cells (V) as a function of the mole fraction of Li (*x*) recorded between 3 and 0.005 V; (a) MgH_2,_ NaH and TiH_2_, (b) LaNi_4_MnH_5,_ Ti_0_._20_V_0.78_Fe_0.02_H_1.55,_ Mg_2_NiH_4_, Mg_2_FeH_6,_ Mg_2_CoH_5,_ TiNiH, and Mg_0.65_Sc _0.35_H_2.25_ [11,79].

Figure 3 shows the experimentally measured potential vs Li^+^/Li^0^ for electrochemical MH*_x_*/Li cells using binary and ternary hydrides. The potential–capacity curves recorded are in agreement with the assumption based on thermodynamics that the conversion reaction is possible for MgH_2_, TiH_2_, NaH and ternary hydrides. These discharge curves correspond to the theoretical reaction MH*_x_* + *x*Li^+^ + *x*e^−^ → M^0^ + *x*LiH and their lengths are in agreement with the number of hydrogen atoms that react with lithium (Figure 3a). For instance the discharge curves of MgH_2_, TiH_2_ [10], NaH involve two and one lithium respectively for two and one hydrogen [11]. Values superior to the number of hydrogen atoms *x* can, however, be reached in relation with either a plateau corresponding to the electrolyte decomposition on carbon at 0.8 V or with a metal alloying reaction at low potentials, especially for Mg (0.17 V vs Li^+^/Li^0^). The potential–capacities curves are lower than the theoretical equilibrium potential due to internal resistance of the cell and are also not totally flat due to kinetic limitations of the system. The equilibrium potential of the MH*_x_*/Li cells can be obtained by galvanostatic intermittent titration (GITT) in open circuit voltage with, for instance, an experimental value of 0.537 V for the MgH_2_/Li cell [11], which is in good agreement with the theoretical value of 0.560 V versus Li^+^/Li^0^ obtained from Nernst law.

Regarding AB*_x_* intermetallic compounds, typical discharge curves obtained from ternary hydrides LaNi_4_MnH_5_, TiNiH [12], bcc Ti_0.20_V_0.78_Fe_0.02_H_1.55_ and Mg_0.65_Sc_0.35_H_2.25_ are presented in Figure 3b. Lengths of discharge curves of *x* = 4.8, 1.2, 1.3, and 2.2 are recorded for LaNi_4_MnH_5_, TiNiH, bcc Ti_0.20_V_0.78_Fe_0.02_H_1.55_ and Mg_0.65_Sc_0.35_H_2.25_ hydrides, respectively. It must be noted that a nice flat plateau is obtained for the AB_5_ compound.

Conversion reactions with lithium ions were also carried out with different complex hydrides based on Mg or Al, especially Mg_2_FeH_6_, Mg_2_CoH_5_ and Mg_2_NiH_4_. These complex hydrides were prepared by reactive grinding [13–14]. They react with lithium ions at average potentials of 0.25, 0.24 and 0.27 V and give discharge capacities of 6.6, 5.5 and 3.6 Li, respectively. Using AlH_3_ [15], Li_3_AlH_6_ [16] or more recently LiAlH_4_, NaAlH_4_ and Na_3_AlH_6_ [17–18] as negative electrode of Li-ion batteries was also reported. It demonstrates the possibility to extend the conversion process to numerous versatile complex hydrides. For these last cases, the discharge curves involve conversion process and alloying reaction, in relation to the close potential of both reaction types.

### II Conversion process reaction mechanisms for hydrides

Reactivity of hydrides with lithium ions predicted from thermodynamic rules and experimentally confirmed for different hydrides is assumed to be a conversion reaction as MH*_x_* + *x*Li → M + *x*LiH. While this general mechanism is obvious, more complex reactions path involving the formation of alloys, solid solutions, metastable or amorphous phases can also be noticed. In the following paragraph reaction mechanisms occurring with MgH_2_ [5,11], TiH_2_ [10], Mg_0.85_Sc_0.65_H_2_ and Mg_2_TMH*_x_* (TM = Fe, Co, Ni, *x* = 6, 5 , 4) [14] hydrides will be described.

#### II.1 Reaction of MgH_2_ with lithium

**Figure 4:**
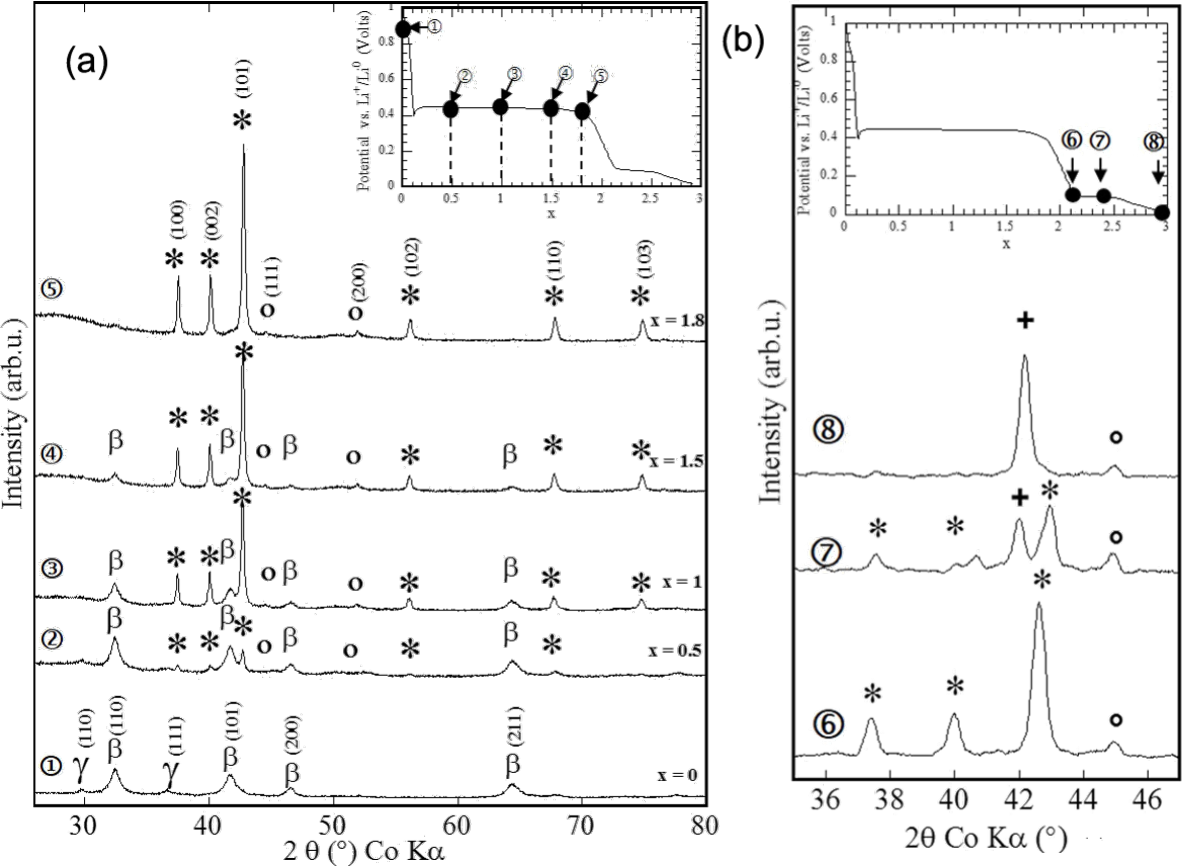
Potential profile and XRD patterns of MgH_2_ electrode at different stages of the conversion reaction. (a) Inset: Evolution of the potential (V) as a function of *x* (mole fraction of Li) for a MgH_2_ electrode cycled between 3 and 0.005 V at a rate of one equivalent of lithium in 100 h. The recorded XRD patterns are associated with the various stages of the discharge, denoted by the numbers (1), (2), (3), (4) and (5) in panel and correspond to *x* = 0 (starting electrode material), *x* = 0.5, 1, 1.5 and 1.8, respectively. The X-ray peaks marked by an asterisk, β, γ and circle correspond to Mg, β-MgH_2_, γ-MgH_2_ and LiH, respectively. b) Inset: Inset: Evolution of the potential (V) as a function of *x* (mole fraction of Li) for a MgH_2_ electrode cycled between 3 and 0.005 V at a rate of one equivalent of lithium in 100 h. The XRD patterns (6), (7), and (8) corresponding to *x* = 2.1, 2.35 and 2.9. The Bragg peaks marked by an asterisk and plus sign correspond to Mg (hcp) and Li (bcc) solid solutions, respectively. Adapted from [5] (copyright 2008 Nature Publishing Group) and [11].

The reaction of Mg hydride with lithium ions is the first example reported in the literature of a Li-driven conversion reaction with hydrides [5,11]. The electrochemical curve recorded at a low cycling rate (one equivalent of lithium in 100 h) during the reaction of MgH_2_ with Li (inset of Figure 4a) shows that the full discharge (length *x* = 2.5 Li) involves two plateaus at 0.44 V and 0.095 V. The XRD patterns, collected at different discharge steps, are presented in Figure 4a. The XRD patterns corresponding to the first plateau (until *x* = 1.8 Li) show a decrease of the intensity of MgH_2_ (tetragonal and orthorhombic phases) Bragg peaks and the appearance of hcp Mg and bcc Li peaks ((100, 002, 101) and (111, 200), respectively). Above *x* = 1.8 (Figure 4b) the first slope observed in the discharge curve corresponds to a shift of the Mg XRD lines in agreement with the formation of a Mg-type solid solution (Figure 4b, no. 6 and Table 1). The last slope corresponds to the formation of a bcc Li-type solid solution (Figure 4b, no. 8), and the plateau to the coexistence of both solid solution types (Li and Mg) (Figure 4b, no. 7). In short, MgH_2_ reacts with Li ions to form Mg and LiH within the conversion process MgH_2_ + 2Li^+^ + 2e^−^ → Mg + 2LiH at around 0.44 V vs Li^+^/Li^0^. Then the freshly formed Mg can react with Li ions at a low potential to form alloys (hcp Mg-type and bcc Li-type solid solutions).

**Table 1:** Lattice parameters for Mg and Mg-type solid solution formed during the reaction of MgH_2_ with Li.

	Mg	Mg hcp solid solution

lattice parameters	*a* = 3.2090(3) Å	*a* = 3.1970(2) Å
	*c* = 5.2100(4) Å	*c* = 5.1410(6) Å

**Figure 5:**
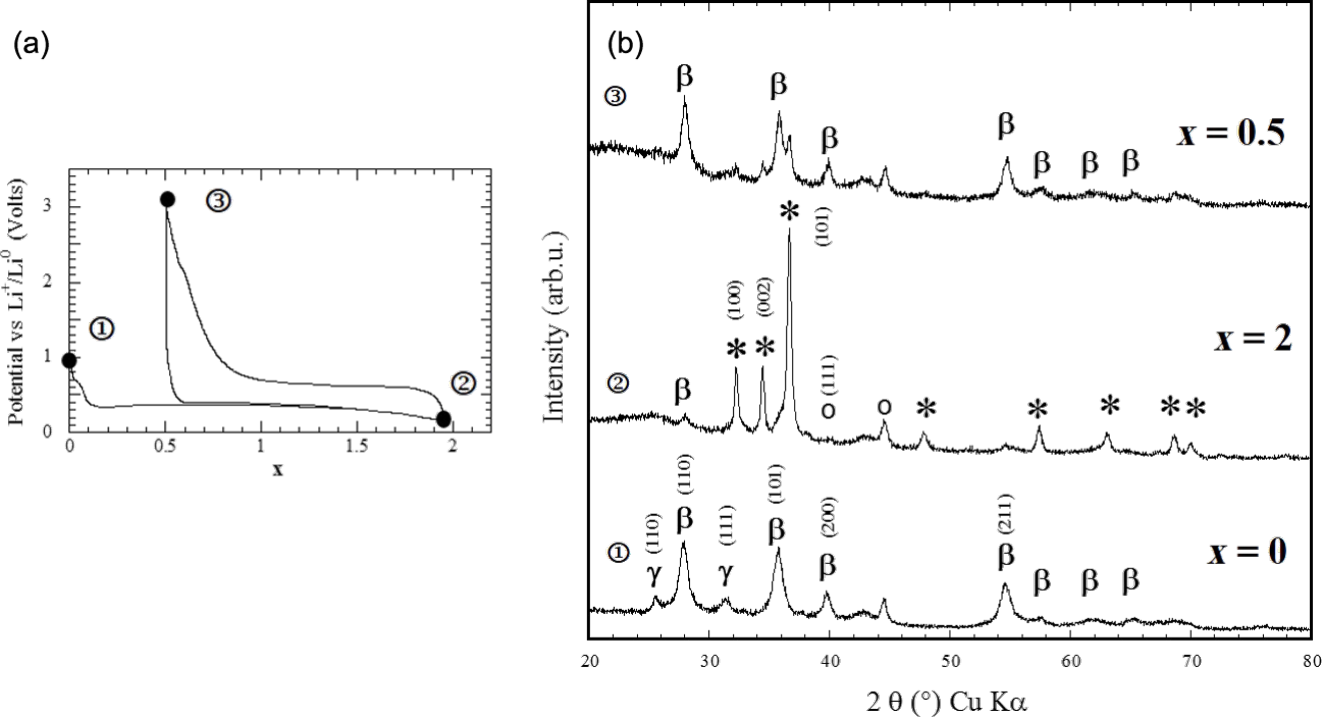
Potential profile and XRD patterns of MgH_2_ electrode at various stages of the conversion reaction. a) Evolution of the potential (V) as a function of *x* for a Li/MgH_2_ cell that was cycled down to *x* = 2 at a rate of one equivalent of lithium in 10 h. b) XRD patterns taken at various stages of the discharge, (1) *x* = 0 (starting electrode material), (2) *x* = 2 (end of the first discharge) and (3) *x* = 0.5 (end of the first charge). The Bragg peaks marked by an asterisk, β, γ, and circle correspond to Mg, β-MgH_2_, γ-MgH_2_ and LiH, respectively. Adapted from [5] (copyright 2008 Nature Publishing Group) and [11].

**Figure 6:**
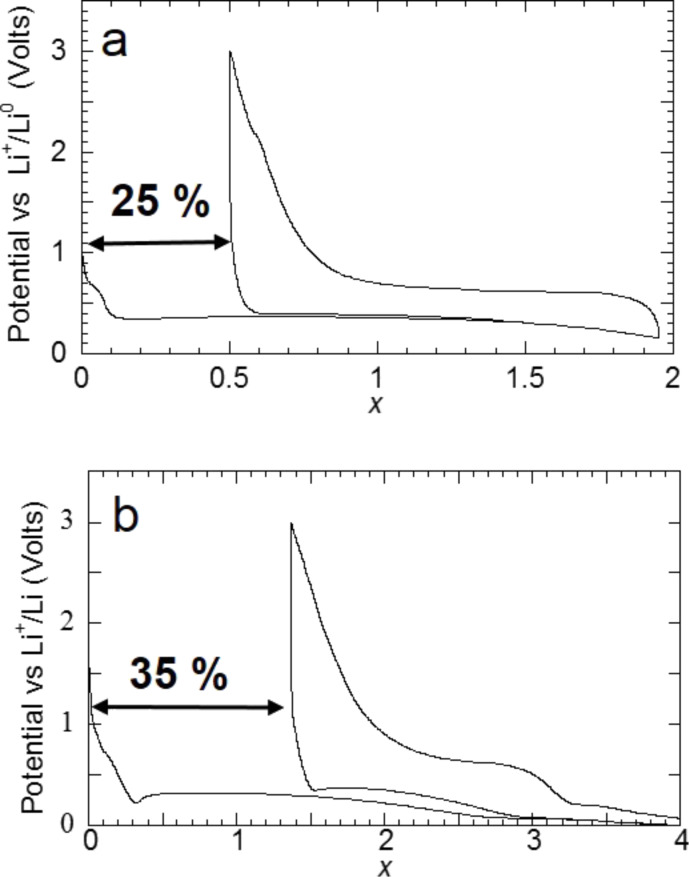
Potential profile of a MgH_2_ electrode at various stages of the conversion reaction. a) Evolution of the potential (V) as a function of *x* for a Li/MgH_2_ cell that was cycled down to *x* = 2 at a rate of one equivalent of lithium in 10 h. b) Evolution of the potential (V) as a function of *x* for a Li/MgH_2_ cell that was cycled down to *x* = 4 at a rate of one equivalent of lithium in 10 h. Adapted from [5] (copyright 2008 Nature Publishing Group) and [11].

Li–Mg alloying reactions can be avoided by limiting the discharge curve to *x* = 2 (Figure 5). In this case a reversible capacity of 1500 mA·h·g^−1^ (irreversible loss of 25%) can be obtained while a reversible capacity of 2700 mA·h·g^−1^ (irreversible loss 33%) is measured for both processes (Figure 6).

#### II.2 Reaction of TiH_2_ with lithium

The study of the reactions of titanium hydride with lithium is motivated by the chemical and structural properties of TiH_2_ [10]. As shown on Figure 1, TiH_2_ is attractive regarding its high theoretical capacities (like all binary hydrides). In addition, an improvement of the conversion process kinetics is expected because of the good electrode conductivity due to the metallic properties of titanium hydride. This reaction can be studied without any parasite reaction as Ti does not form alloys with lithium.

**Figure 7:**
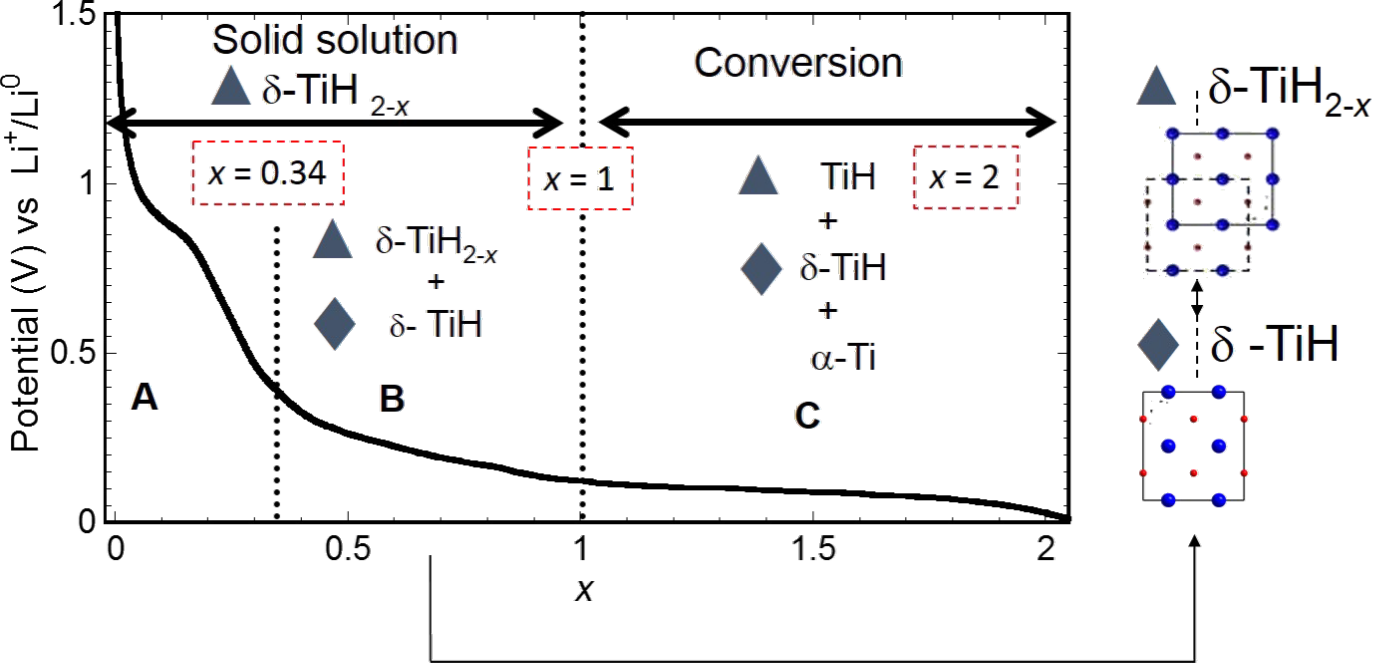
Summary of the dehydrogenation process of TiH_2_ electrode δ-TiH_2−_*_x_* (fcc): black triangles, TiH (fco): black diamonds, projection along the *b*-axis of δ-TiH_2−_*_x_* (fcc): black triangles and TiH (fco): black diamonds showing the relationship between fcc (black triangles) and fco (black triangles) structures. Adapted from [10] (copyright 2012 Elsevier) and [11].

The TiH_2_ discharge capacity, presented in Figure 7, drastically differs from that of MgH_2_ through the presence of two slopes prior to a pseudo plateau. XRD analyses of the electrode during electrochemical discharge show that the reaction of TiH_2_ with Li involves three steps. The two first slopes correspond to the formation of an fcc δ-TiH_2−_*_x_* solid solution until *x* = 0.34 (first slope) that transforms partially from *x* = 0.34 to 1 in a distorted face-centered orthorhombic phase δ-TiH (fco) (second slope). Note that from *x* = 0.34 to 1, the peritectic transformation: hcp α-Ti(H) → fcc δ-TiH_2−_*_x_* + δ-TiH explains hexagonal close-packed (hcp) α-formation absence. Finally, conversion process occurs in the pseudo plateau with the formation of hcp α-Ti and LiH.

#### II.3 Reaction of Mg_2_MH*_x_* with lithium

**Figure 8:**
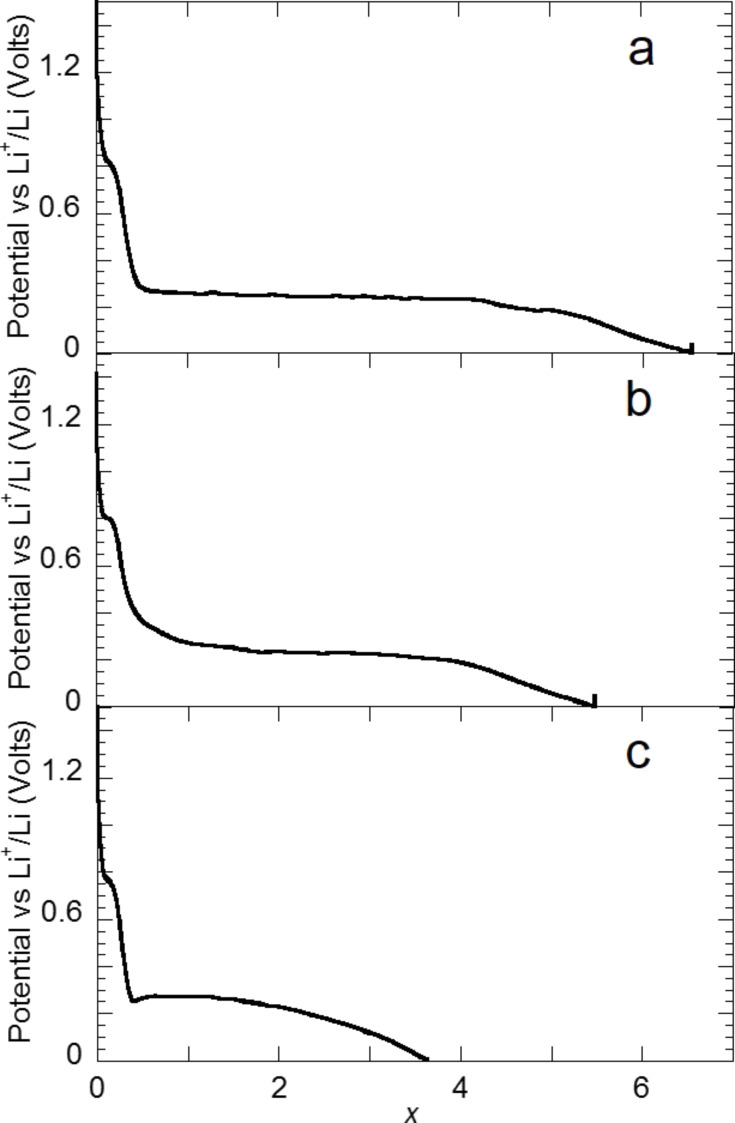
Discharge curves for a) Mg_2_FeH_6_, b) Mg_2_CoH_5_ and c) Mg_2_NiH_4_ electrodes prepared by reactive grinding as a function of Li (*x*) mole fraction, recorded at a current rate of one equivalent of Li in 10 h. Adapted from [13] (copyright 2013 Elsevier) and [19].

**Figure 9:**
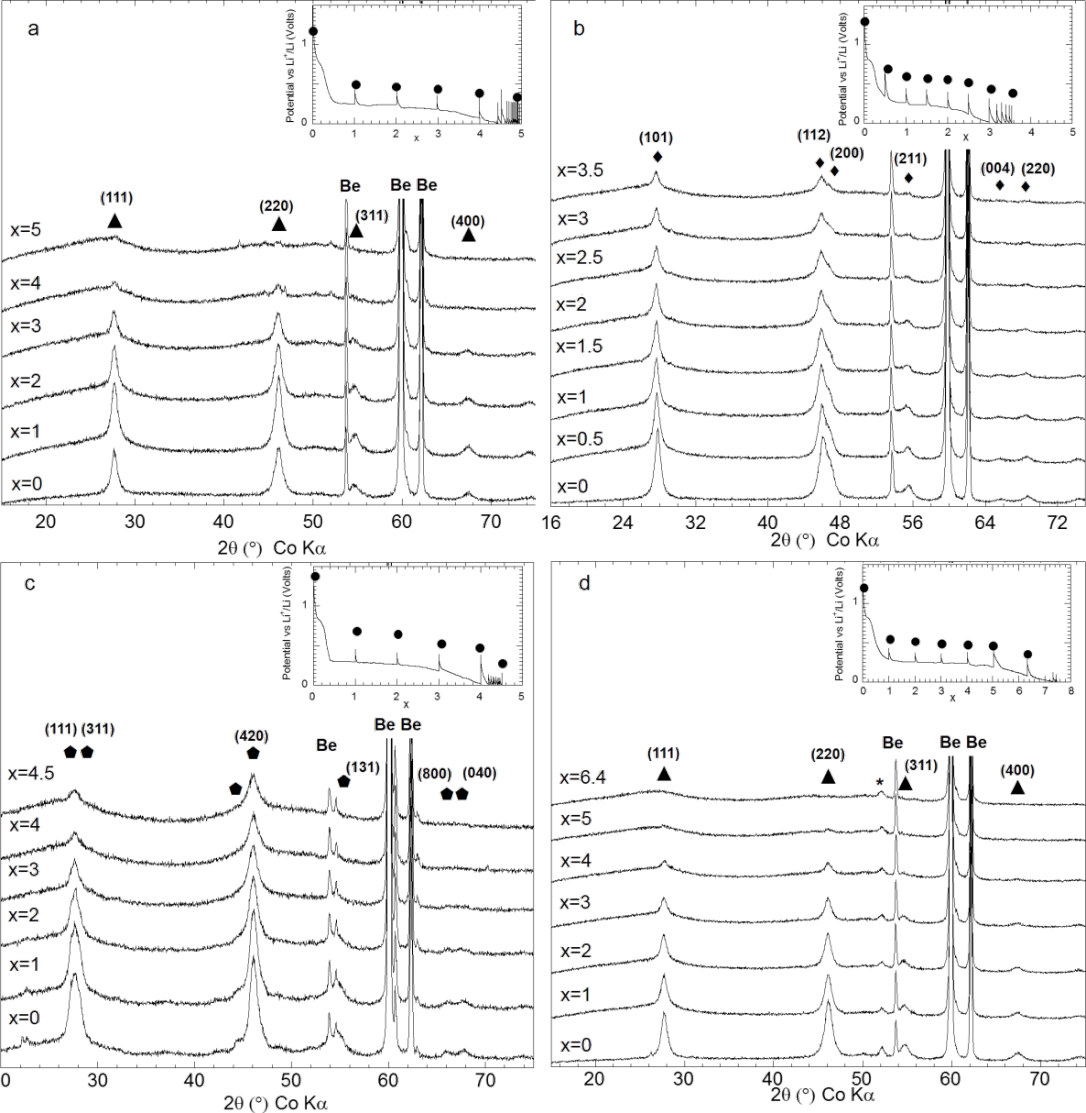
In situ XRD patterns of a) Mg_2_FeH_6_, b) Mg_2_CoH_5_ and c) Mg_2_NiH_4_ and d) Mg_2_FeH_6_-10% Ct,*z* electrodes prepared by reactive grinding as a function of the mole fraction of Li (*x*) Inset: Potential profile of a) b) c) and d) obtained during GITT (rate: one equivalent of Li in 10 h, relaxation time: 10 h). Adapted from [13] (copyright 2013 Elsevier) and [19].

After studying the reaction of titanium hydride with lithium, during which a reaction path involving the formation of the metastable fco δ-TiH phase occurs, the complex hydrides Mg_2_FeH_6_, Mg_2_CoH_5_, Mg_2_NiH_4_ were chosen as models system for a conversion process with high energy storage capacities and unusual thermodynamics properties [13,19]. In fact, the decomposition of Mg_2_FeH_6_ and Mg_2_CoH_5_, which is expected during their electrochemical reaction with lithium, can be used for the formation of a conductive Fe or Co matrix, which is helpful to reverse the reaction between Mg and LiH. In addition, the far-from-equilibrium electrochemical process is an interesting tool to search for new intermetallic compounds consisting of Mg and Fe or Co [20–21]. As shown in Section I.3, Mg_2_MH*_x_* (M = Fe, Co, Ni , *x* = 6,5,4) react with lithium at roughly the same potential (around 0.25 V vs Li^+^/Li^0^) and the capacities measured during the discharge are close to the theoretically obtained values (Figure 8). From a structural point of view, a common behavior can be noticed for the reaction of lithium with all complex hydrides from the XRD characterizations of the electrodes. A complete (for the case of Mg_2_FeH_6_) or partial disappearance of the parent phases is observed, which occurs without any formation of metals (Figure 9). This loss of crystallinity suggests the formation of an electrode with nanocrystalline or amorphous structure. The formation of nanoscale Fe during the decomposition of Mg_2_FeH_6_ is confirmed by XAS and Mössbauer spectroscopy [22]. Ex situ XAS spectroscopy of the Mg_2_CoH_5_ and Mg_2_NiH_4_ electrodes revealed the formation of disordered MgCo and Mg_2_Ni intermetallic compounds.

The intensity reduction of the XRD lines, which occurs without broadening, involves shifts of the lattice parameters. For instance, theMg_2_CoH_5_ lattice parameters *a* and *c* rise until *x* = 1 (from 4.4940(3) to 4.517(2) Å and from 6.582(1) to 6.608(1) Å, respectively; a cell volume expansion of 1.44%), evoking a phase transformation. For Mg_2_NiH_4_ the lattice parameter *c* decreases from 6.538(1) to 6.477(2) Å, which is concomitant with a phase transformation involving a low-hydrogen-content hydride for *x* ≤ 3: Mg_2_NiH_4_ → Mg_2_NiH [23–24].

The reaction mechanism can therefore be summarized as follows:


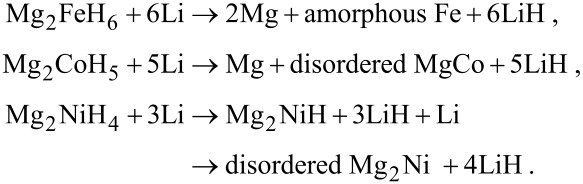


The reaction with Mg_2_FeH_6_ during the conversion process with lithium ions is the first example for the production of an amorphous phase “2Mg + Fe”.

#### II.4 Reaction of other Mg-based hydrides with lithium

The reactions of 2MgH_2_ + M (M = Cu, Si) and 0.65Mg + 0.35M (M = ScH_2_, Ti) mixtures prepared by reactive grinding under 90 bar of hydrogen pressure with lithium ions were also studied [19,25–27]. The electrochemical behavior of MgH_2_ is not affected by the presence of a second element, Cu or Si, and significant reversible capacities for the conversion process (>1000 mA·h·g^−1^) are obtained. In the case of the mixture 2MgH_2_ + Si, an additional capacity below 0.2 V vs Li^+^/Li^0^ due to the alloying reaction of Si with Li is observed. This combined conversion (MgH_2_)/alloying (Si) system presents the highest theoretical capacity anode with the possibility to reach approximately 6000 mA·h·g^−1^.

The production of pure Mg_0.65_Sc_0.35_H_2.25_ (1900 mA·h·g^−1^) by reactive grinding from magnesium and scandium hydride (MgH_2_ + ScH_2_) is not possible. Instead, a mixture of 68% of Mg_0.65_Sc_0.35_H_2.25_, 20% of MgH_2_ and 12% Mg_2_FeH_6_ is obtained. The formation of Mg_2_FeH_6_ is due to the strong abrasion of the grinding tools through scandium hydride. The discharge curves of this hydride mixture involves 2.25Li at 0.32 V vs Li^+^/Li^0^ and the corresponding X-ray diffraction pattern obtained at the end of the discharge shows the presence of both Mg_0.65_Sc_0.35_H_0.8_ and Mg_0.65_S_0.35_H_1.5_ cubic phases in agreement with the results obtained in KOH electrolyte and reported by Notten et al. [28].

Such desirable electrochemical behavior is also obtained with the Mg_0.65_Ti_0.35_H_2.25_ mixture prepared by reactive grinding with a reversible conversion process involving both MgH_2_ and TiH_2_ without the addition of carbon. As expected, TiH_2_ increases the conductivity of the electrode and a full discharge process is obtained without carbon. The development of such Mg-based system appears to be a promising opportunity.

### III Kinetics limitations of hydrides for conversion reactions: example of MgH_2_

The huge numbers of hydrides reported in the literature exhibit different structures, electronic properties and thermodynamics stabilities. Using the hydrides classification proposed by Libowitz et al. [29–30] metallic, covalent and ionic hydrides can be identified.

Given the fact that the electric behavior is an important parameter for the electrochemical reaction, the issue of the poor electric conductivity of ionic and iono-covalent hydrides must be solved. For instance, the ionic hydride MgH_2_ exist as α, β and γ, with the space groups *P*4_2_/*mnm* (tetragonal, *a* = *b* = 4.516 Å, *c* = 3.020 Å) *Pa*−3 (cubic) and *Pbcn* (orthorhombic, *a* = 4.526 Å, *b* = 5.448 Å, *c* = 4.936 Å), respectively. The tetragonal phase is the more stable phase. These hydrides exhibit band gap energies of 5.3, 5.6 and 4.2 eV, respectively, and are not electronic conductors but insulators [31].

**Figure 10:**
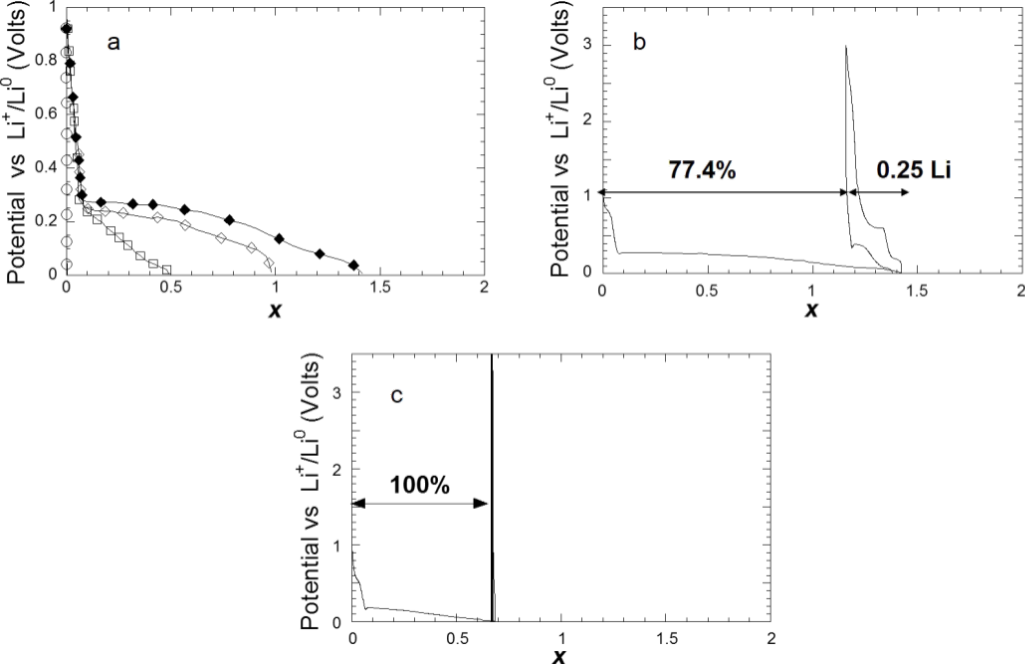
The evolution of the potential (V) as a function of *x* (mole fraction of Li) for a MgH_2_ electrode prepared with commercial hydride cycled between 3 and 0.005 V. a) Discharge curves at a rate of one equivalent of lithium in 100 h as function of the content of Super p carbon of the electrode: open circles: 0%, open squares: 15%, open diamonds: 20%, black diamonds: 25%. b) Discharge–charge curve at a rate of one equivalent of lithium in 100 h for a content of 25% of Super P carbon. c) Discharge–charge curve at a rate of one lithium in 10 h for a content of 25% of Super P carbon at a rate of one lithium in 10 h [11].

Figure 10 shows the poor electrochemical reactivity of commercially available tetragonal MgH_2_ vs Li^+^/Li^0^, with no electrochemical capacity during the first discharge. The addition of an electronic conductive material, such as the graphite Super P (electronic conductivity: 10^3^ S·cm^−1^) increases the electronic conductivity of the electrode. As presented in Figure 10a, the discharge capacity of the hydride increases with the amount of graphite. The addition of 25% of graphite gives a discharge capacity of 1.4Li for 2 h for a current of one mole of electron in 100 h. The contribution of Super P carbon to the total capacity remains very small and the maximum of the contribution that can be reached is less or equal to *x* = 0.25.

Note that the conductivity of the electrode is not only influenced by MgH_2_ but also by conducting metallic Mg and insulating LiH, which are generated during the reaction. The improvement of the poor electronic conductivity of the active material in powder form has been widely addressed in the literature for different electrode materials (in aqueous and non-aqueous electrolytes). A solution is carbon-coating through chemical or physical methods. For electrochemical reactions carried out in thin films in KOH electrolyte with non-conductive hydrides such as MgH_2_, or Mg_2_NiH_4_, the presence of a small amount of non-hydrogenated compound (few percent), as Mg or Mg_2_Ni, in the starting material is sufficient to produce a satisfactory electronic conductivity inside the electrode and the addition of carbon is not necessary. Another issue consists in the production of ternary hydrides films with Mg, Ti and H [32–33]. The metallic behavior TiH_2_ counters the insulating influence of MgH_2_.

#### III.1 Influence of the particle size on the reversibility of the conversion process

Regarding the reversibility of the conversion reaction, the poor capacities obtained during the first charge for electrodes composed of commercial MgH_2_ (0Li and 0.25Li for a current rate of one equivalent of Li in 10 and 100 h, respectively) clearly show than the benefit of the addition of Super P carbon to the electronic conductivity of the active material seems completely lost when the reaction is inversed (Figure 10b,c).

Other parameters than the conductivity of the active material also govern the efficiency of the conversion process. In fact, the volume variation of the electrode during the conversion process MH*_x_* + *x*Li^+^ + *x*e^−^ → M^0^ + *x*LiH drastically affects the conductivity between the particles. For MgH_2_ the volume variation is 83% between MgH_2_ (61.59 Å^3^) and Mg (46.46 Å^3^) + 2LiH (2 × 33.3 Å^3^). Thus, during the discharge the electrode volume increases with the lithium transfer and decreases during the lithium extraction. As a consequence, voids are created inside the electrode and disconnect the particles from each other and from the current collector at the same time. Decreasing the particle size is one way to accommodate for the volume variation of the electrode and to maintain cohesion of the interfaces and the connection between particles and the current collector. Reducing the length of diffusion for Li and H can be helpful for volume accommodation and preserving interfaces. Different approaches to reduce the particles sizes and to accommodate the volume variation of the electrode can be considered and will be presented in the following paragraph: Effect of grinding on commercial hydride, effect of three hydrogen sorption, effect of grinding commercial hydride with carbon, effect of activation by sorption cycles follow by the grinding with carbon, reactive milling.

#### III.2 Effect of mechanical grinding on commercial MgH_2_

**Figure 11:**
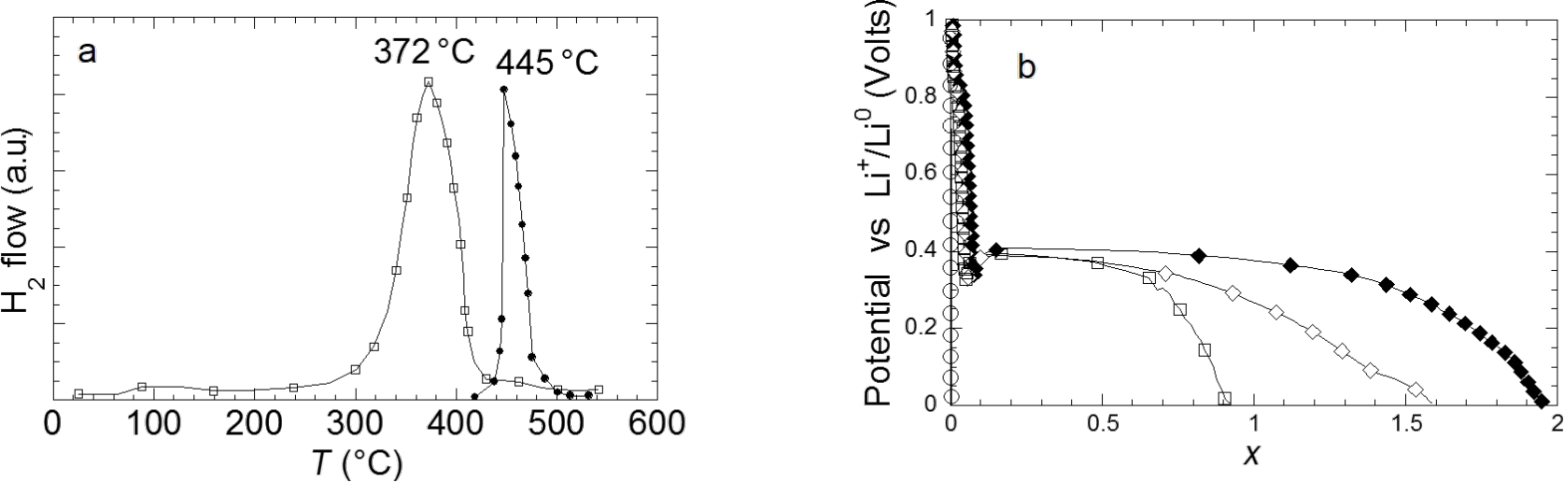
a) DSC traces of commercial MgH_2_ unground and ground for 20 h. b) Evolution of the potential (V) as a function of *x* (mole fraction of Li) for a MgH_2_ electrode prepared with commercial hydride ground for 20 h in the Spex mixer mill and cycled between 3 and 0.005 V at a rate of one equivalent of lithium in 100 h. Super P carbon content (%) of the electrode: open circles: 0%, open squares: 15%, open diamonds: 20%, black diamonds: 25% [11].

**Figure 12:**
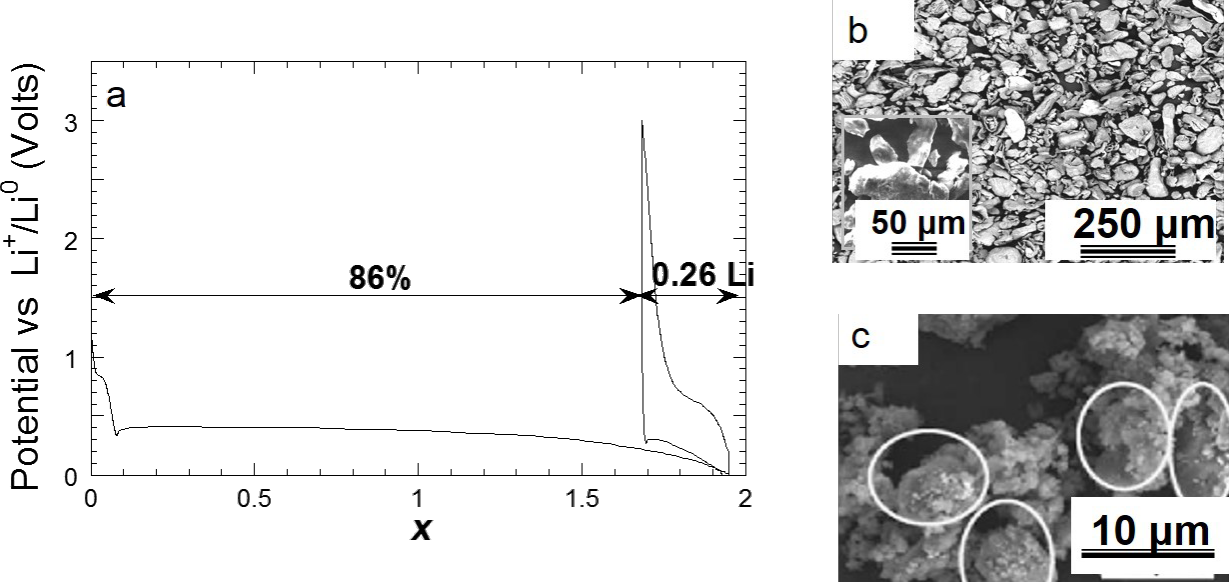
a) Evolution of the potential (V) as a function of *x* (mole fraction of Li) for a MgH_2_ electrode prepared with commercial hydride, ground 30 h in a Spex mixer mill and cycled between 3 and 0.005 V at a rate of one equivalent of lithium in 100 h. b) SEM image of a MgH_2_ commercial powder ground 30 h in the Spex mixer mill. c) SEM image of MgH_2_ commercial powder ground 30 h in the Spex mixer mill showing the agglomeration of particles [11].

Grinding of commercial MgH_2_ enables a faster hydrogen desorption with a desorption maximum at 372 °C compared to 445 °C for the untreated commercial material (Figure 11a). As expected, discharge efficiency increases with the amount of Super P carbon added to the electrode (Figure 11b). In this case, a full discharge capacity of *x* = 1.95Li can be achieved when 25% of graphite is added. The improved performance of the ground sample is due to a reduction of the crystallite size down to few nanometers, which facilitates the diffusion of hydrogen and lithium by increasing the number of diffusion paths. However, despite an improvement of the discharge kinetic, the reversibility of the hydride is hardly changed (Figure 12a). The reversible capacity of ground commercial MgH_2_ after 30 h of grinding is actually *x* = 0.26Li.

The particle size cannot be reduced below 0.1 μm through grinding, because immediate agglomeration of smaller particles occurs (Figure 12b,c). So, even if crystallite size of few nanometers can be reached during grinding, the formation of agglomerates of 5 to 30 μm (consisting of primary particles of 0.1–5 μm, Figure 12b) limits the reversibility of the conversion process.

#### III.3 Effect of hydrogen sorption cycles on MgH_2_

The particle size of the hydride is also reduced through decrepitation during hydrogen desorption–absorption cycles. This solid–gas reaction not only reduces the particle size of the hydride but also enhances its reactivity vs Li-ions. In the case of the system Mg/MgH_2_, 5% of Super P carbon was mixed with the Mg powder in order to increase the thermal conductivity of the powder and to prevent the necking of particles during sorption cycles.

**Figure 13:**
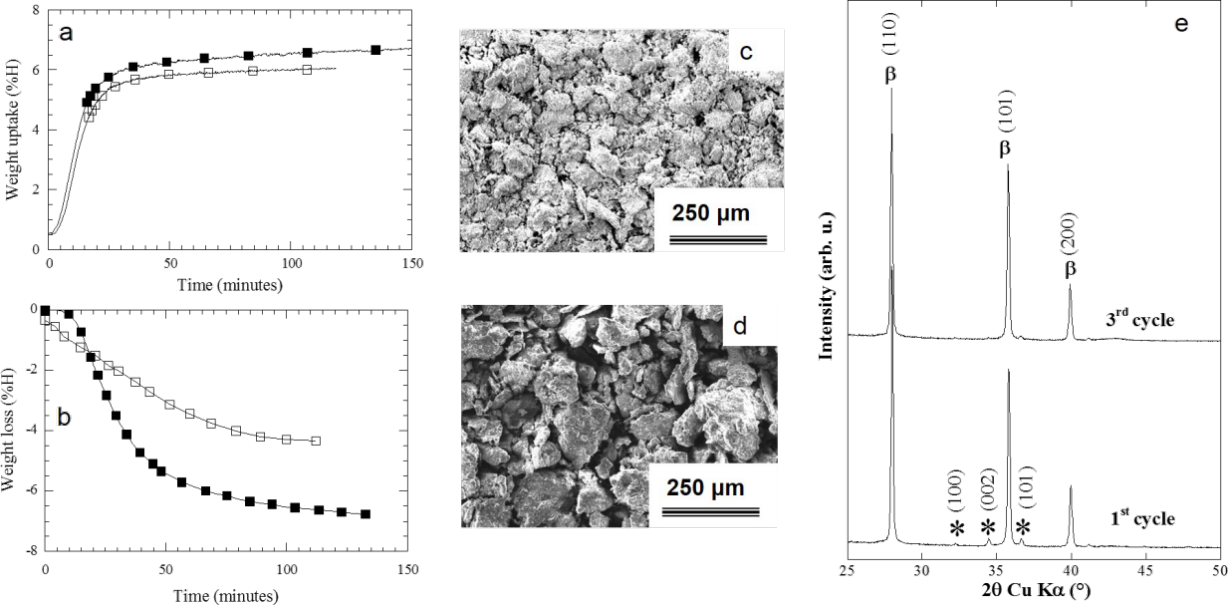
a) Absorption and b) desorption kinetics at 350 °C for Mg–10%C_10_*_,_*_320_ composite (open squares): first cycle, black squares: third cycle; c) SEM image of the MgH_2_ powder after one absorption; d) SEM image of the MgH_2_ powder after three absorptions; e) XRD patterns of the MgH_2_ powder after one and after three absorptions [11].

Figure 13a and Figure 13b show the hydrogen sorption kinetics at 350 °C for Mg/MgH_2_ for the first and the third cycles (uptake and loss in wt % hydrogen). Hydrogen sorption kinetics and capacities increase from the first to the third cycle and then are constant for the subsequent cycles (not shown here). This activation process observed during the first three cycles can be correlated with a particle size reduction (Figure 13c and Figure 13d) and an increase of the BET surface area from 7 to 14 m^2^·g^−1^ while no change of the hydride crystallinity is observed (Figure 13e).

**Figure 14:**
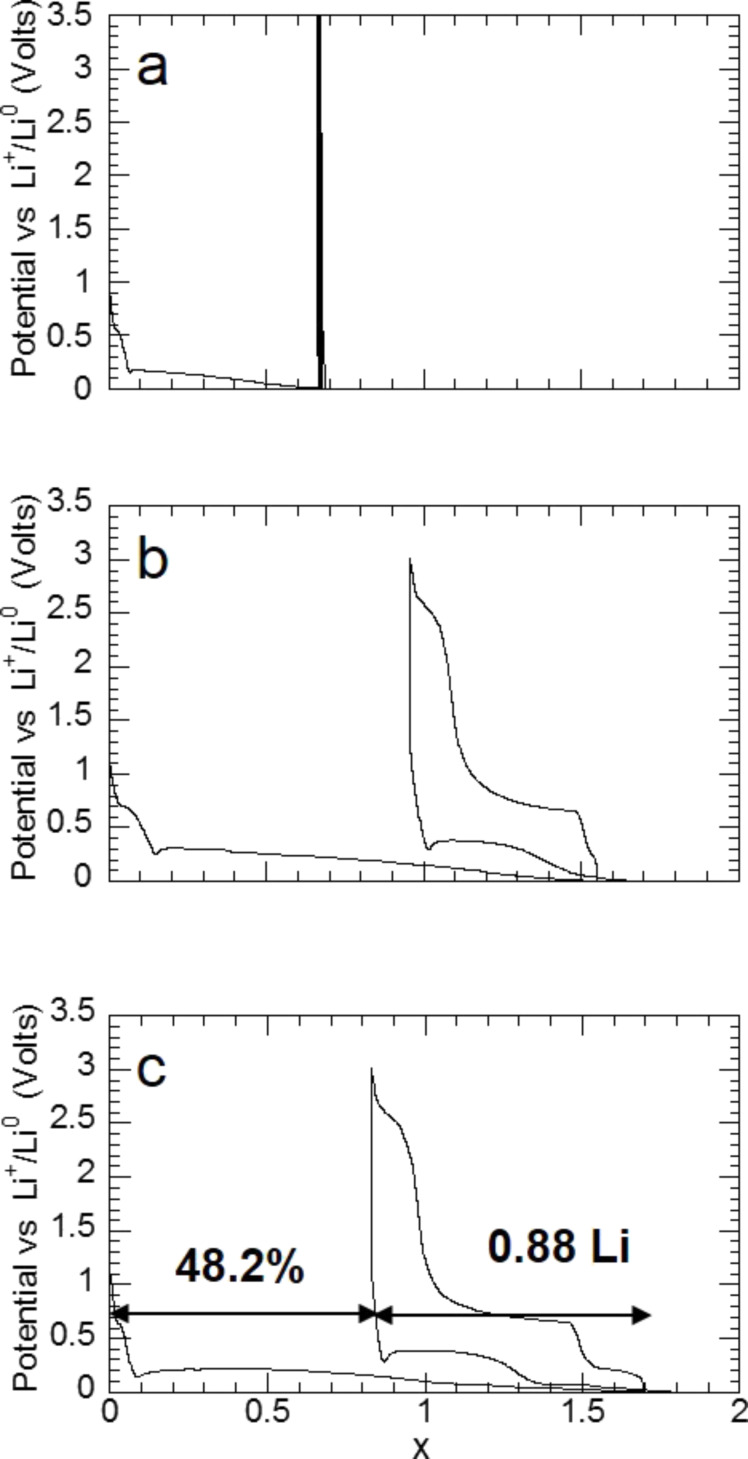
Evolution of the potential (V) as a function of *x* (mole fraction of Li) for MgH_2_ electrodes cycled between 3 and 0.005 V at a rate of one equivalent of lithium in 10 h. a) MgH_2_ commercial hydride; b) MgH_2_ obtained after one absorption of hydrogen; c) MgH_2_ obtained after three absorptions of hydrogen [11].

The effect of three sorption cycles on the reaction of MgH_2_ with lithium (Figure 14) shows that reversible capacity drastically increases compared to that of ground commercial hydride and reaches 0.88Li after three sorption cycles (Figure 14c). Despite an attractive increase of reversible capacity, an irreversible loss (48%) can be noticed on potential–capacity curves.

#### III.4 Effect of grinding of MgH_2_ with carbon

Grinding of commercial MgH_2_ with a pre-ground C*_t_*_,_*_z_* carbon, where *t* refers to the pre-grinding time and *z* to the carbon BET surface area, was used to enhance the efficiency of the conversion reaction. Grinding of commercial MgH_2_ with C*_t_*_,_*_z_* carbon is supposed to create of a porosity volume inside the electrode, corresponding to a volume increase due to matter transfer. This porous volume is then recovered during the lithium extraction and the total volume change of the electrode is then minimized. In addition, the carbon also acts as conductive additive and a coating agent, which prevents the agglomeration of the hydride particles during grinding. A detailed study of the effect of mechanical milling on the physical/chemical and electrochemical properties compared to AB_5_ alloys is available in [34].

**Figure 15:**
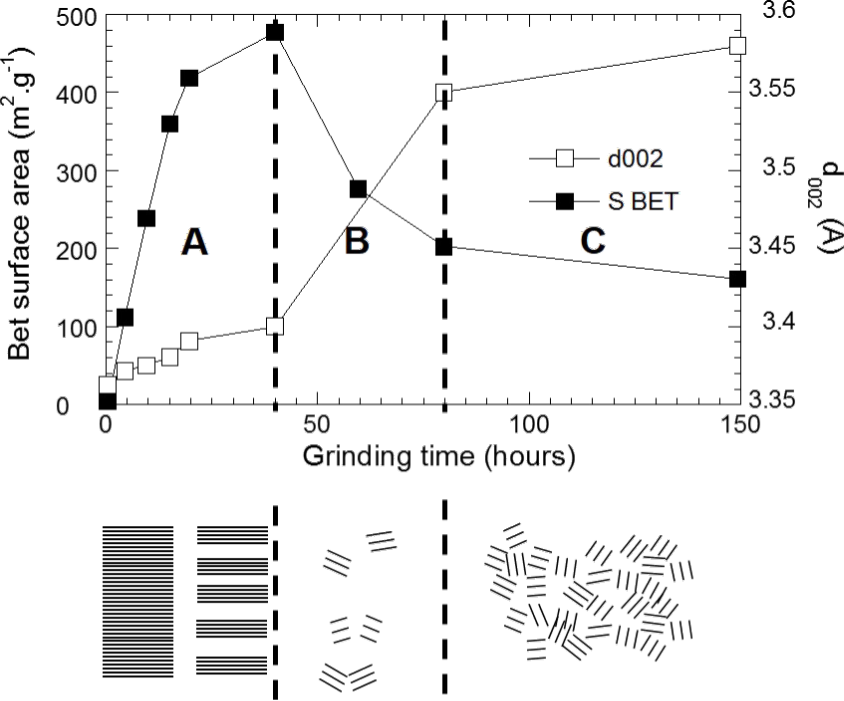
Graphite BET surface area (m^2^·g^−1^) and *d*(002) interlayer distance (Å) as a function of grinding time (h) [11].

Figure 15 shows the evolution of the BET surface *z* (in m^2^·g^−1^) and of the *d*(002) interplanar spacing of carbon C*_t_*_,_*_z_*, as a function of the milling time *t* (in h). Two main grinding steps can be noticed in Figure 15. First the BET surface area increase while *d*(002) remains almost constant (step A) and then the BET surface area decrease and *d*(002) drastically increases (step B). Firstly, The mechanical energy transferred to the carbon produces an exfoliation of the graphene layer. Then, the cumulated mechanical energy coming from the grinding is sufficient to promote fissure propagation within the graphene layer, resulting in the fracture of the C–C covalent bonds, leading to the formation of very reactive edge carbon atoms and unstable particles which agglomerate together. The degree of disorder for carbonaceous materials increases with increased milling time and is proportional to the *d*(002) distance, as previously established [35–36]. Note that the C-free bonds created during the fracture of the graphene layer serve as oxygen scavengers, and their agglomeration and coating of the alloy particles enable a better chemical/physical protection against oxidation [34].

**Figure 16:**
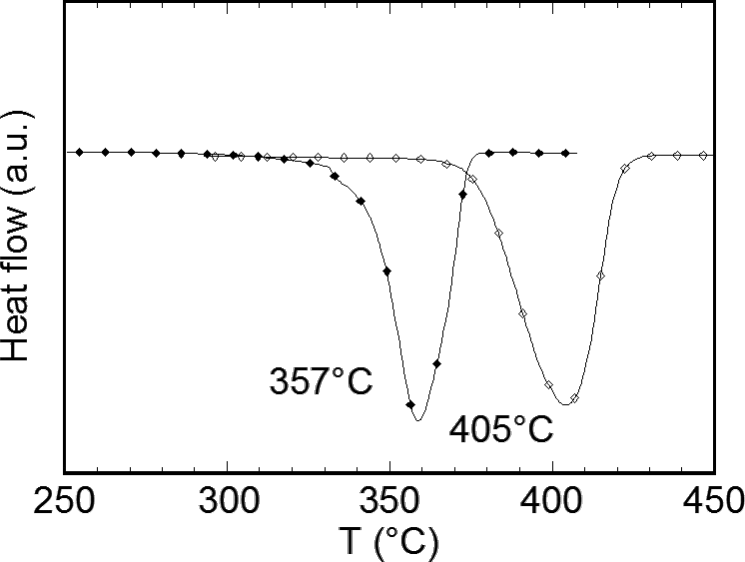
Thermodesorption of commercial MgH_2_ (black diamonds) and commercial MgH_2_ ground 4 h with 10% of C*_t_*_,_*_z_* carbon (open diamonds) [11].

Based on the milling behavior of carbonaceous material [34–35], MgH_2_ is ground using a carbon having the maximum BET surface area in order to agglomerate carbon particles on MgH_2_ particles. DSC traces of MgH_2_–10% C*_t_*_,_*_z_* composite obtained after 4 h of grinding shows a decrease of 48 °C of the desorption peak maximum of hydride carbon composite compared to the commercial hydride, as expected (Figure 16).

**Figure 17:**
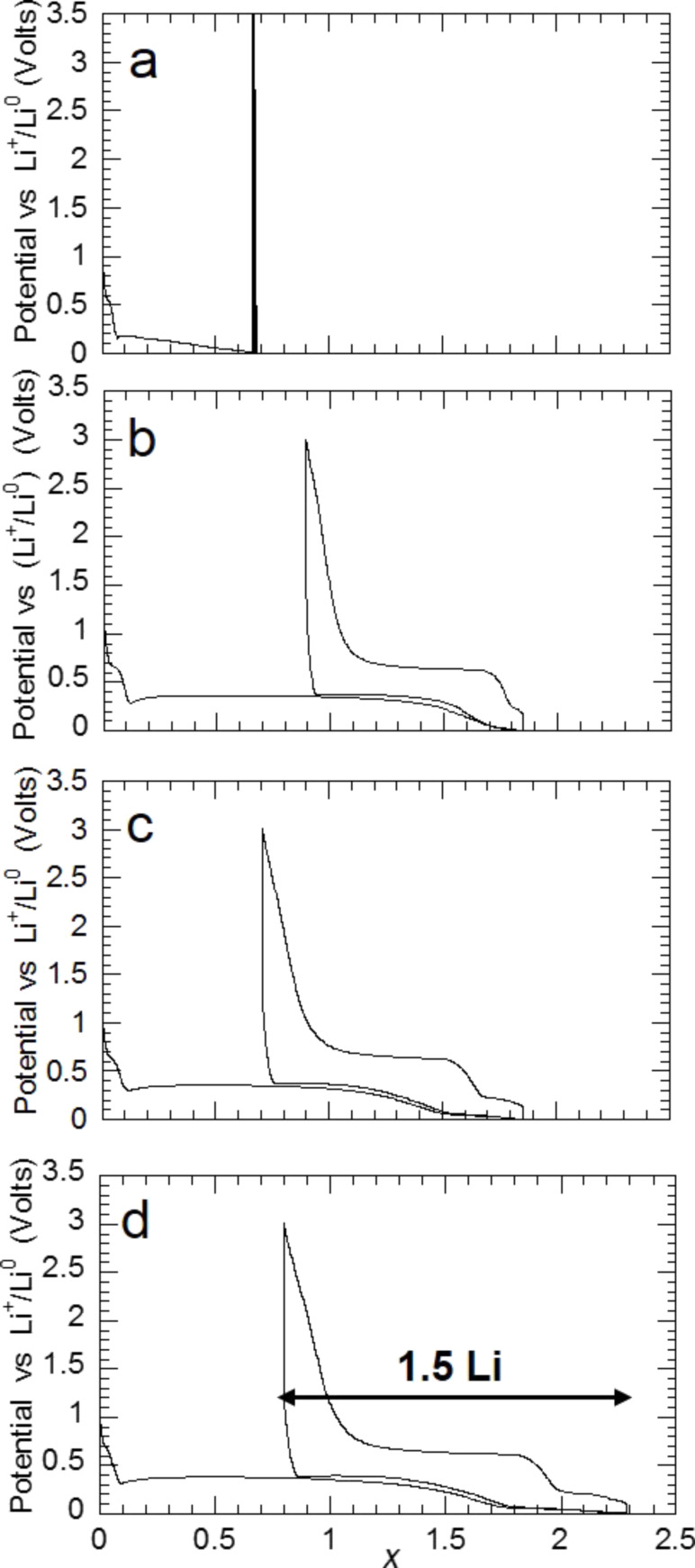
Evolution of the potential (V) as a function of *x* (mole fraction of Li) for MgH_2_ electrodes cycled between 3 and 0.005 V at a rate of one equivalent of lithium in 10 h. a) MgH_2_ commercial hydride; b) MgH_2_ commercial hydride ground for 4 h with 10% of C*_t_*_,_*_z_* carbon; c) MgH_2_ after one absorption of hydrogen and then ground for 4 h with 10% of C*_t_*_,_*_z_* carbon; d) MgH_2_ after three absorptions of hydrogen and then ground for 4 h with 10% of C*_t_*_,_*_z_* carbon [11].

The dispersion of the hydride particles into carbon increases the thermal conductivity of the powder and helps the hydrogen release. With regard to the electrochemical properties, the potential–capacity curves of an electrode composite of MgH_2_–10% C*_t_*_,_*_z_* obtained after 4 h of grinding shows a spectacular enhancement of the reversible capacity with 0.96Li (= 1000 mA·h·g^−1^) for an irreversible loss of 48% (Figure 17b).

**Figure 18:**
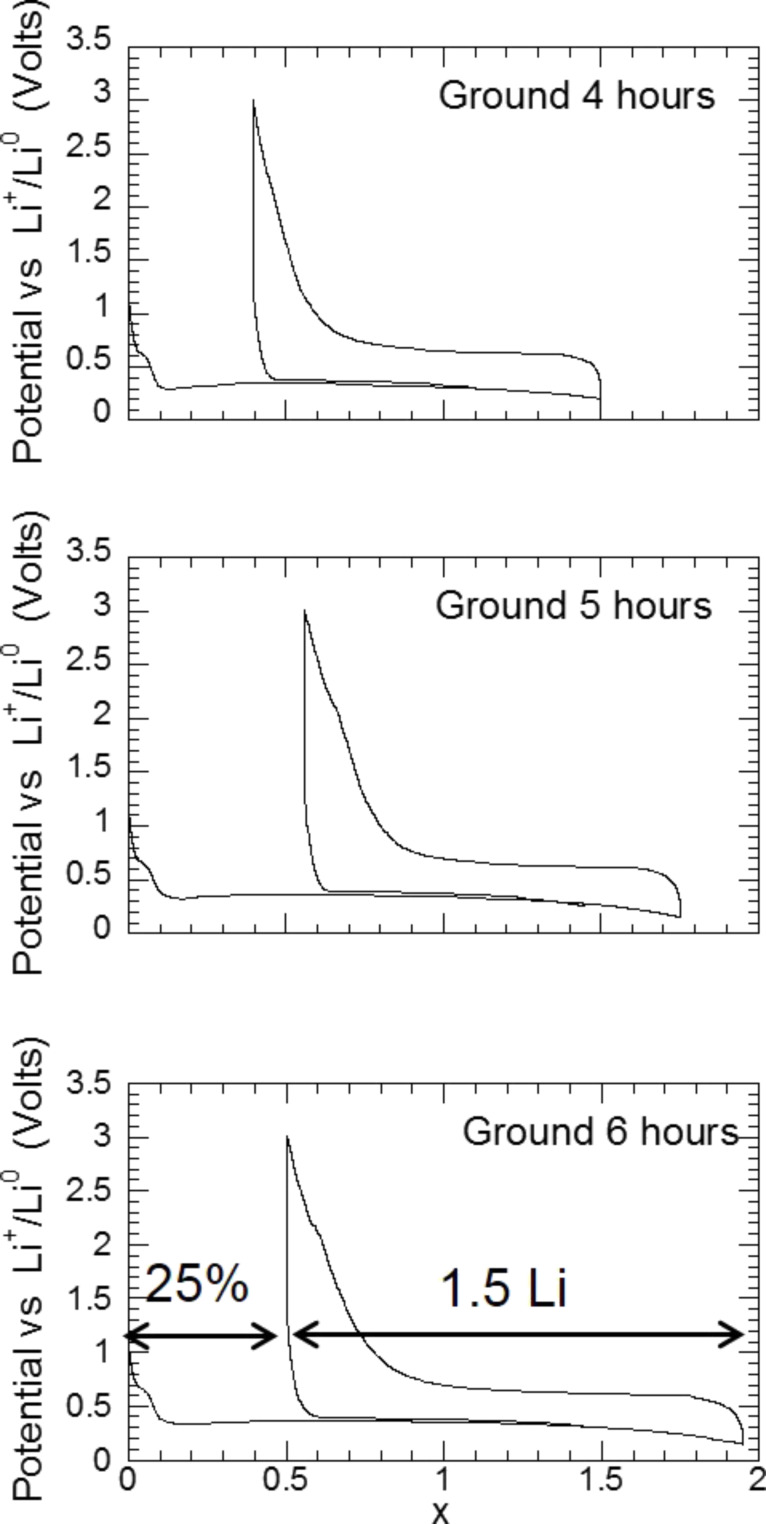
Evolution of the potential (V) as a function of *x* for a Li/MgH_2_ cell that was cycled down to 0.15 V vs Li^+^/Li^0^ at a rate of one equivalent of lithium in 10 h. (MgH_2_ is obtained after three absorptions of hydrogen and then ground 4, 5 or 6 h with 10% of C*_t_*_,_*_z_* carbon) [11].

The grinding of MgH_2_ with carbon was also carried out with MgH_2_ that was activated through three sorption cycles. A synergic effect, involving both the hydride activation by solid–gas reaction and grinding with a C*_t_*_,_*_z_* carbon to enhance the reversibility was noticed (Figure 17c,d). For instance, a capacity of 1500 mA·h·g^−1^ for an irreversible loss of 35% after three sorption cycles and 4 h of grinding with 10% of C*_t_*_,_*_z_* carbon was obtained (Figure 17d). This reactivity enhancement enables to obtain interesting reversibilities, free of alloying reaction (with a cut of voltage of 0.15 V vs Li^+^/Li^0^). When the grinding time of the activated MgH_2_ + C*_t_*_,_*_z_* mixture varied from 4 to 6 h, a reversible capacity of 1480 mA·h·g^−1^ for an irreversible loss of 25% is thus obtained at 0.15 V vs Li^+^/Li^0^ (Figure 18).

#### III.5 Effect of reactive milling under hydrogen

Reactive milling under hydrogen constitutes a powerful method for the synthesis of hydrides with the advantage to grind and to hydrogenate the sample in one single step. Applied to MgH_2_, a subsequent grinding step with carbon C*_t_*_,_*_z_* is needed to obtain similar electrochemical performances than for the sample prepared by three hydrogen sorption steps followed by grinding with the carbon C*_t_*_,_*_z_* (1600–1700 mA·h·g^−1^ at 0.005 V vs Li^+^/Li^0^ for 6 h of grinding with carbon).

#### III.6 Effect of metal catalyst addition to the MgH_2_ carbon composite

The improvement of the sorption kinetics of MgH_2_ through catalyst addition (i.e., transition metals [37–38] transition metal oxides [39–41] and halides [42]) has been widely studied in the literature. Nb_2_O_5_ is one of the most efficient catalysts [43] enabling fast hydrogen desorption kinetics with 7.6 wt % of hydrogen desorbed in 100 s at 300 °C. To improve the conversion reaction of MgH_2_ with lithium, the addition of a metal catalyst, which is more suitable than oxides in relation with the electrode conductivity, has been reported by Nakayama et al. in a patent [44–45]. A charge–discharge efficiency of 93.9% for the conversion reaction with MgH_2_ is reported by the grinding addition of 3 atom % of a nickel catalyst (particle size 20 nm) in the hydride MCMB carbon mixture. In this last case the irreversible loss can be drastically reduced to 7% for a reversibility capacity of 2608 mA·h·g^−1^ at 0.01 V vs Li^+^/Li^0^ and a current rate of C/50.

### IV Performance improvements of hydride-based electrodes

Different samples preparation methods for MgH_2_–18% C*_t_*_,_*_z_* (activation by three hydrogen sorption steps or reactive milling followed by grinding with C*_t_*_,_*_z_* carbon) are able to produce an hydride carbon composite electrode with full discharge capacity and 75% reversibility (1500 mA·h·g^−1^ at 0.15 V vs Li^+^/Li^0^) during the first charge for a current of one equivalent of electrons in 10 h. Despite this improvement of the electrode reversibility of the MgH_2_ carbon composite, the cycle life is however limited due to the 83% volume variation, leading to an electronic interparticular conduction loss. Moreover, at a high current rate, the slow hydrogen motion leads to a limitation of the reversible capacity. The influence of the current rate and of the number of electrochemical cycles on the reactivity of the MgH_2_ carbon composite will be described in the two following paragraphs.

#### IV.1 Influence of the current rate on the electrode reactivity: example of MgH_2_

**Figure 19:**
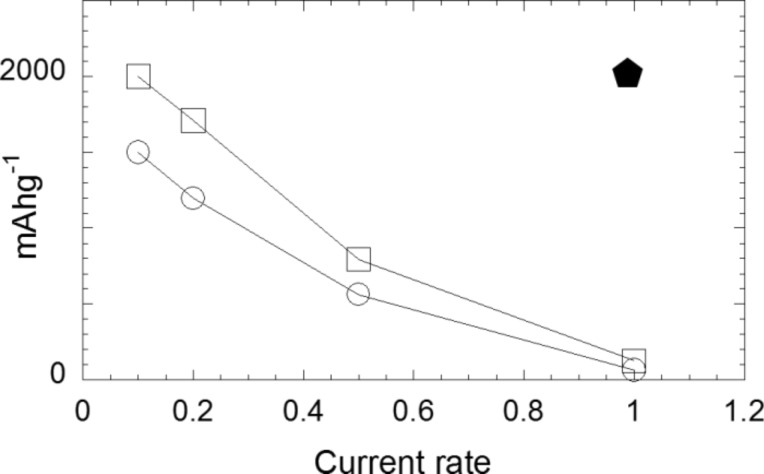
Capacities of a MgH_2_ electrode obtained after three absorptions of hydrogen and then ground for 4 h with 10% of C*_t_*_,_*_z_* carbon as a function of the current rate (number of Li exchange per hours) open squares: discharge capacity, open circles: charge capacity; black pentagon: discharge capacity of a MgH_2_ thin film of 200 nm [11].

The experimental capacity decreases when the current rate increases and, for an exchange rate of one equivalent of electrons in one hour, the capacity is close to zero. This result shows that the reactivity improvement with different sample preparation methods is still insufficient for real application requiring electrode power and fast charge (Figure 19). However, for a thin film of MgH_2_ (200 nm) (prepared by R. Griessen group at the Vrije Universiteit Amsterdam) a full discharge capacity of 2Li can be obtained for a current of one equivalent of electrons in one hour. This fact definitively confirms than the reduction of the diffusion distances is the key to achieve high power electrodes with hydrides for conversion reaction.

#### IV.2 Effect of polymer binders on MgH_2_-based electrode cycle life

The electrochemical cycling behavior of MgH_2_–18% *C_t_*_,_*_z_*/Li was studied and compared with that of MgH_2_–33.3% CMC–33.3% C*_t_*_,_*_z_*/Li and MgH_2_–33.3% CMC-f–33.3% C*_t_*_,_*_z_*/Li cells where CMC and CMC-f are polymer binders (sodium carboxymethylcellulose and sodium carboxymethylcellulose-formate, respectively [46]). CMC [47–48], which was already widely investigated for silicon-based electrodes [49–60], surprisingly sustains the 270% volume change taking place during cycling of the Si-based electrode. Beattie et al. [54] estimated the CMC binder and carbon quantity needed to fill the holes created during lithium extraction from the Si alloy electrode to be around 66%. It was experimentally confirmed that large capacities and long cycle lives of the electrodes are obtained for a 33% Si/33% CMC/33% C mixture. This ratio of active material/binder/carbon was chosen to improve the electrode cycle life of MgH_2_. In addition to CMC, a derivative binder with a formyl ester group was tested (CMC-f) [61].

**Figure 20:**
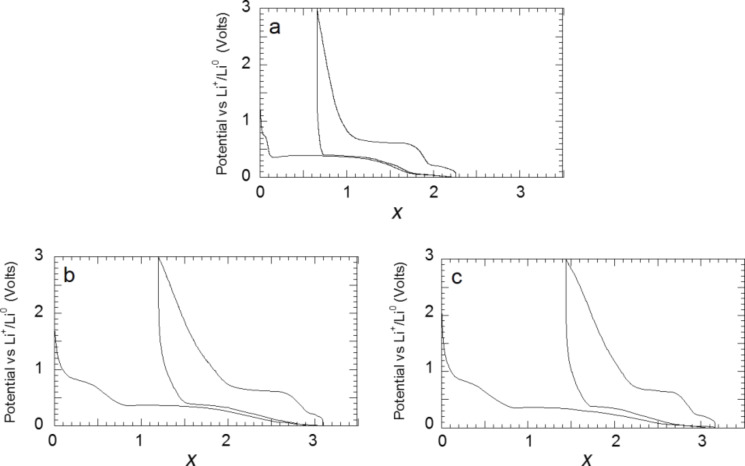
Evolution of the potential (V) as a function of *x* (mole fraction of Li) for MgH_2_ electrodes cycled between 3 and 0.005 V at a rate of one equivalent of lithium in 10 h. (a) MgH_2_–18% C*_t_*_,_*_z_*; (b) 33.3% MgH_2_–33.3% CMC–33.3% C*_t_*_,_*_z_*; (c) 33.3% MgH_2_–33.3% CMC-f–33.3% C*_t_*_,_*_z_*. Adapted from [46]. Copyright 2011 Elsevier.

**Figure 21:**
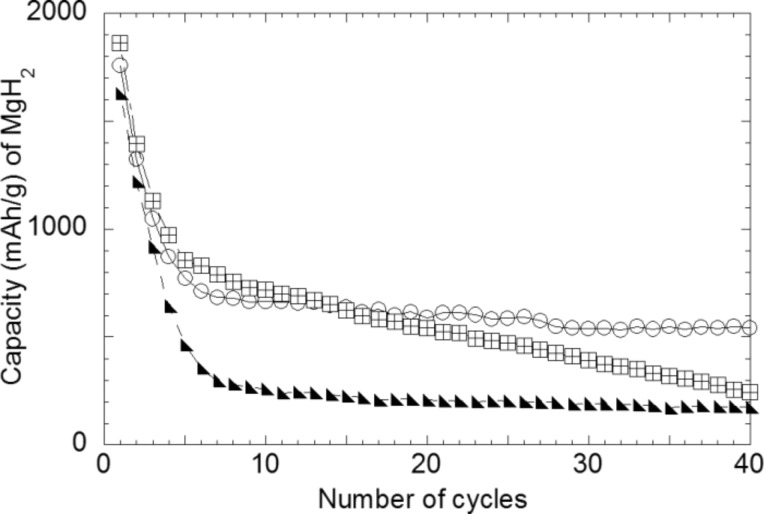
Electrochemical cycling performance for MgH_2_ composite electrodes: MgH_2_–18% C*_t_*_,_*_z_* (black triangles), 33.3% MgH_2_–33.3% CMC–33.3% C*_t_*_,_*_z_* (open squares) and 33.3% MgH_2_–33.3% CMC-f–33.3% C*_t_*_,_*_z_* (open circles). Cycling between 3 and 0.005 V, rate of one equivalent of lithium in 10 h. Adapted from [46]. Copyright 2011 Elsevier.

MgH_2_–18% C*_t_*_,_*_z_*/Li, MgH_2_–33.3% CMC–33.3% C*_t_*_,_*_z_*/Li and MgH_2_–33.3% CMC-f–33.3% C*_t_*_,_*_z_*/Li discharge–charge curves are shown in Figure 20. It appears there that high reversible capacities are preserved independently of the amount of active material additives in the electrode. Indeed, reversible capacities of 1700 mA·h·g^−1^, 1800 mA·h·g^−1^ and 1900 mA·h·g^−1^ are obtained for the MgH_2_–18% C*_t_*_,_*_z_*/Li (Figure 20a), MgH_2_–33.3% CMC–33.3% C*_t_*_,_*_z_*/Li (Figure 20b) and MgH_2_–33.3% CMC-f–33.3% C*_t_*_,_*_z_*/Li (Figure 20c) cells, respectively. An increase of the carbon content from 18 to 33% leads to an increase of the irreversible loss (from 25 to 39%) due to electrolyte decomposition at the carbon surface. The reversible capacities of the MgH_2_ electrodes as a function of the cycle number are presented in Figure 21 (MgH_2_–18% C*_t_*_,_*_z_*/Li, MgH_2_–33.3% CMC–33.3% C*_t_*_,_*_z_*/Li and MgH_2_–33.3% CMC-f–33.3% C*_t_*_,_*_z_*/Li). From theses curves it is clear that the presence of the CMC-type binder enhances the cycle life of the electrode. While a capacity value of 174 mA·h·g^−1^ is obtained for the electrode without binder, 240 mA·h·g^−1^ and 542 mA·h·g^−1^ are obtained for CMC and CMC-f binders after 40 cycles, respectively. The weak capacity, which originates from carbon (20 mAh·g^−1^ after 40 cycles) is not at the origin of the better capacity retention of the MgH_2_–33.3% CMC–33.3% C*_t_*_,_*_z_*/Li and MgH_2_–33.3% CMC-f–33.3% C*_t_*_,_*_z_*/Li electrodes. Thus, porosity created by the polymers might explain the better volume accommodation of the electrode during lithium extraction. Further studies are needed for a better understanding of the nature of the polymeric interactions with carbon and metal hydride and their role during the solid mixing of CMC-type binders with MgH_2_ and C*_t_*_,_*_z_*.

#### IV.3 Role of interface and particle size on reversibility of the conversion reaction: example of TiH_2_

The reaction of titanium hydride (TiH_2_) with lithium ions previously described involves a reaction path that can be summarized as follows:

*x* ≤ 0.34: δ-TiH_2_(fcc) + 0.34Li → δ-TiH_1.66_ + 0.34LiH,

0.34 < *x* ≤ 1: δ-TiH_2−_*_x_* + *x*Li → δ-TiH(fco) + *x*LiH,

1 < *x* ≤ 2: δ-TiH_2−_*_x_*(fcc) + δ-TiH(fco) + Li → α-Ti(hcp) + LiH.

**Figure 22:**
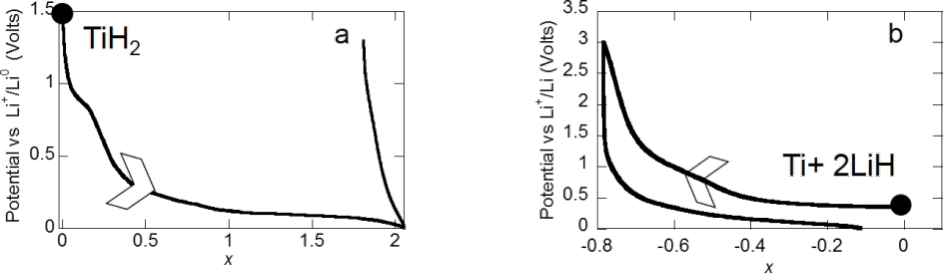
Potential profile of a) TiH_2_ electrode ground for 5 h with 10% of C*_t_*_,_*_z_* carbon b) Ti + 2LiH electrode as a function of *x*, the mole fraction of Li (rate one equivalent of Li in 100 h). Adapted from [6]. Copyright 2009 Elsevier.

Within this conversion process, a full discharge capacity of 1072 mA·h·g^−1^ is obtained for the TiH_2_ ground with 10% of C*_t_*_,_*_z_* carbon for 5 h. However, while this reaction is free of any alloying reaction with lithium, no electrochemical reversibility was noticed during electrode charge (Figure 22a). To support the idea that interfaces and particles sizes play a key role on conversion reaction reversibility with titanium hydride, the electrochemical behavior of a (2LiH + Ti) composite was studied [6]. This composite was prepared through a mechanochemical reaction between TiH_2_ and metallic Li. The capacity of the first charge reached 455 mA·h·g^−1^ whereas after one cycle the reversible capacity was still 428 mA·h·g^−1^, corresponding to a capacity retention of 94% (Figure 22b). The XRD pattern collected at the end of the first charge indicated the formation of titanium hydride in agreement with the theoretical conversion reaction: 2LiH + Ti → TiH_2_ + 2Li^+^ + 2e^−^. This is a clear example showing that when thermodynamics allow a conversion reaction to occur, interfaces and particles size constitute the pertinent parameters that governs the process reversibility.

#### IV.4 Synthesis of nanoscale composite hydrides: a perspective to achieve all solid state batteries

It must be emphasized first that production of hydride nanoparticles by reactive milling seems to be an unsuccessful route as long as the agglomeration of the freshly fractured particles is not controlled. Cryomilling could be a potential solution to avoid metal welding and to produce material on the laboratory scale, but this will require significant technological improvements to become economically viable for large scale material production.

Chemical methods, such as encapsulation or confinement strategies used in the design of energy storage and conversion materials, also constitute new synthetic routes that have shown promising results [62–63]. Thus, for solid-state hydrogen storage applications, very fast hydrogen absorption/desorption kinetics have been indeed confirmed for nanoscale Mg hydride (MgH_2_) confined into the porosity of different carbon hosts [64–65] or chemical matrices [66–67]. Very recently, composites containing MgH_2_ nanoparticles (with a narrow size distribution of 1–10 nm) which were well-dispersed into a porous carbon host have been prepared by Zlotea et al. [68]. These were produced with varying metal content up to 50 wt % and were designed to be used as a negative electrode for Li-ion batteries. These composites show interesting electrochemical behavior, especially regarding their cycle life stability (500 mA·h·g^−1^ after 40 cycles) and have a stable nanoparticle size distribution during electrochemical cycling.

Other promising materials, produced through a physical vapor transport deposition method such as Mg/MgH_2_ nanowires or nanofibers are under development for a few years now [69–71]. For instance Mg nanowire shows interesting modifications of both thermodynamics and kinetics compared to the bulk material: a decrease of the dissociation energy of about 12%, (30–50 nm nanowires: 65.3 kJ·(mol H_2_)^−1^ bulk material: 74 kJ·(mol H_2_)^−1^).

Hydriding chemical vapor deposition (HCVD) is also a powerful method to produce in situ high purity nano/microscale MgH_2_ under hydrogen. This method, which enables to play with temperature and pressure, is a nice tool for the preparation of a variety of particles having controlled size and shapes (nanofibers, nanoparticles, microdendrites, irregular bulk, hexagonal microplates and microspheres). A good picture of this synthesis method, leading to the production of tailored materials, is given by a pressure–temperature diagram, similar to the diagram of Nakaya et al. [72]. Mass production and applications of such materials in the field of hydrogen storage and batteries technologies will be an interesting challenge for the next decade.

#### IV.5 Use of computational methods to look for better materials

Computational methods can also offer interesting alternatives to help the search and development of materials for hydrogen storage and batteries. The idea is, for instance, to look for materials having high volume capacity, minimal expansion volume and high lithium mobility. A recent study by molecular dynamics simulations has revealed that the Li diffusion in MgH_2_ nanocluster doped with Fe, Ni, Ti or V is independent of the presence of the transitions metal [73]. The metals improve the hydrogen desorption kinetics. Again, this example shows the mutual interest of this kind of device for hydrogen storage and batteries technologies.

The search for the existence of new stable hydrides in the Mg–Li–H system was also addressed by several groups through density functional theory approach (DFT) [74–75]. Ternary hydrides in the system Li–Mg–H, such as Li_2_MgH_4_ and LiMgH_3_, are insulators dominated by ionic bonds. Their preparation from Li, Mg and H_2_ is energetically favorable, but may be kinetically inhibited by separation into pure phases. The effects of various light-metal (Mg, Al, Li) and transition-metals (V, Cr, Mn, Fe, Co, Cu, Zn) dopant on the electrochemical properties of NiTiH hydrides as anodes for Li-ion batteries where theoretically studied by Qian et al. [76–77] with Al, Cr, Mn and Fe being the most promising according to the authors.

## Conclusion

Studies of the reaction of hydrides with lithium ions started with the pioneer work with MgH_2_, where lithium-driven conversion reactions were firstly demonstrated for the metal hydride family. The study was subsequently extended to other metal and complex hydrides, especially TiH_2_ and AlH_3_, and is in now in progress, particularly because of the important number of different potential interesting compounds [78]. Metal hydrides present the advantage of having high capacities in a safe potential window of 0.1–1 V vs Li^+^/Li^0^. Moreover, these hydrides show the lowest polarization reported to date for conversion reaction electrodes, as compared to oxides, sulfides, nitrides, phosphides and fluorides compounds, and therefore, constitute promising candidates for negative electrodes in Li-ion batteries.

This research field focuses now mainly on nanocomposite synthesis in order to enhance the limited electrochemical cycling performances, the main drawback of hydrides. Electrode technology is, thus, the next challenge, considering the design of the current collector, the preparation of polymeric binders and the mixing of hydride and electrolyte together with careful studies to achieve better capacity retention performance or to scale up the nanocomposite elaboration process. On the other hand, the results obtained at the present time emphasize the importance of the knowledge of fundamentals aspects to control the conversion complex process where reaction paths, interfaces and particle size are the keys parameters. Use of hydrides as anode for Li-ion batteries needs also strong interactions between batteries and fuel-cell communities to be wholly fruitful [79]. In the final analysis, hydrides as a new concept for negative electrodes bridges Li-ion battery and hydrogen storage technologies together and can constitute a promising opportunity for the discovery and the achievement of new energy storage technology for the next 20 years.
